# Experimental Study on the Chemical Composition, Microstructure, Heat Treatment and Mechanical Properties of Steels for Special Knife Applications

**DOI:** 10.3390/ma18214900

**Published:** 2025-10-26

**Authors:** Jaroslava Svobodová, Miroslav Müller, Ludmila Nováková, Josef Hořejší

**Affiliations:** 1 Faculty of Engineering, Czech University of Life Sciences, Kamýcká 129, 165 00 Prague-Suchdol, Czech Republic; muller@tf.czu.cz; 2 Faculty of Mechanical Engineering, J. E. Purkyne Universty in Usti nad Labem, Pasteurova 3334/7, 400 96 Usti nad Labem, Czech Republic; 3 Independent Knife Maker

**Keywords:** special knife steels, chemical composition, microstructure, heat treatment, mechanical properties, structure–property relationships, SEM/EDS analysis, powder metallurgy, laminated steels, forge welding

## Abstract

This study presents an experimental investigation of steels used in special knife applications, focusing on the interrelationship between chemical composition, microstructure, heat treatment, and mechanical properties. Four representative materials were analysed: VG10 (stainless steel with nickel-laminated edges and a VG10 core), RWL_34_^TM^ (powder-metallurgical steel), laminated steel K110+N695 (with a nickel interlayer), and forge-welded steel K600+K720. The steels were characterised using OES, optical microscopy and SEM, supported by EDS for local chemical analysis. Microhardness testing was applied to individual structural regions to correlate carbide morphology, layer interfaces, and heat-treatment response with hardness values. The results reveal pronounced differences in structural homogeneity and defect occurrence. Powder-metallurgical RWL_34_^TM^ exhibited the most uniform microstructure with finely dispersed Cr carbides, achieving high hardness and absence of structural defects. In contrast, laminated and forge-welded steels contained large primary carbides, carbide precipitation at grain boundaries, porous cavities, and insufficient cohesion in interlayers or weld zones, which may compromise toughness. VG10 and K110+N695 showed carbide coarsening caused by inadequate heat treatment, whereas K600+K720 revealed weld-related defects and heterogeneous phase structures. Overall, the study demonstrates the critical role of heat treatment and processing route in determining blade quality and performance. The findings provide guidance for optimising steel selection and processing technologies in advanced cutlery engineering.

## 1. Introduction

The knife is among the oldest technical tools used by humans. Since prehistoric times, it has represented a fundamental instrument ensuring survival in an environment full of natural threats, while simultaneously becoming a universal tool for everyday activities.

Cutlery production represents a very narrow segment of metal tool manufacturing, which is relatively small in both volume and industrial importance. Precisely for this reason, however, the development of knife steels relies heavily on the knowledge and research results from related and more extensive fields, particularly toolmaking. Cutting and forming tools are subjected to requirements comparable to those of knife blades—they must exhibit high hardness, long edge retention, toughness, and, in many cases, corrosion resistance [1,2]. This enables the effective transfer of scientific and technical knowledge from the field of tool steels directly into cutlery practice.

One of the critical challenges in high-performance knife steels is controlling eutectic carbides, especially large M_7_C_3_ and M_23_C_6_ carbides, which tend to precipitate during solidification. These carbides concentrate in interdendritic regions, disrupting the continuity of the matrix and causing stress concentrations, which reduce both toughness and processability. This problem is extensively studied in stainless and tool steels, where controlling carbide size and distribution is key to optimising edge performance and resistance to fracture [3].

Moreover, microalloying with elements like Ti or Nb promotes the formation of finer MC carbides that are more evenly distributed and thermally stable [4]. This leads to a significant reduction in the detrimental eutectic carbides, thereby enhancing the overall performance of blade steels.

Historical steelmaking technologies differed fundamentally from present-day methods [5]. While modern metallurgy allows for the production of highly pure and homogeneous materials, traditional forge-welded steel contained a significant amount of slag inclusions. In some cases, however, these impurities were deliberately exploited as elements positively influencing the mechanical properties of the final material. The demanding nature of production led to steel being regarded in the past as an exceptionally valuable material.

Systematic knowledge of the processes occurring in steel began to develop only in modern times, particularly with the advancement of analytical methods capable of observing microstructure and its influence on material properties [5]. Nevertheless, in the scientific literature, the issue of traditional steels and their use in cutlery is represented only to a limited extent, as the main attention is usually devoted to final products rather than the material aspects themselves.

A significant example of historically and technologically remarkable materials is Damascus steel. This category includes both the original Wootz type and later variants produced by forge welding. Both types represent valuable subjects of study due to their combination of unique mechanical properties and aesthetic appearance.

Modern research into carbide formation has revealed that the cooling rate during solidification and the degree of micro segregation are key to controlling carbide morphology. Faster cooling rates refine dendrite structures and reduce the size of interdendritic carbides. Conversely, slower cooling near the centre of large ingots promotes the growth of coarse, network-like carbides, which are detrimental to edge performance [3]. Controlling carbide precipitation is not only a matter of cooling rate but also chemical composition. The presence of elements such as Nb, V, or Ti strongly influences the formation of MC-type carbides. These carbides tend to be blocky or skeletal, and when too large, they resist dissolution and act as crack initiators. Recent research recommends microalloying and composition optimisation to limit the size and frequency of detrimental carbides in steels used for fine cutting tools [3].

Ultra-high-carbon steels with globularized carbides provide an ideal balance [6] between wear resistance and toughness for blade applications.

Post-solidification techniques, such as electroslag remelting (ESR), significantly reduce segregation and stabilise the microstructure [7], making them promising for cutlery-grade steels.

Research in the field of tool steels provides a wealth of findings directly applicable to cutlery. Particularly essential for linking disciplines such as cutlery and toolmaking are steels such as D2, AISI 440C, 154CM, or modern powder-metallurgical variants like CPM S30V and CPM S90V. Studies confirm that the synergy of microalloying (e.g., Mo, V) and tailored heat treatment protocols enables steels like C PM S30V to simultaneously achieve high hardness (>60 HRC) and fracture toughness, critical for blade longevity [2,8].

The historically oldest method of joining steels, based on a diffusion process at high temperatures and pressure, is forge welding. In its manual form, it relies on the hammer and the skill of the blacksmith, while in industrial production, it employs power hammers, rolling, or pressing, which allow for greater control of the conditions [9].

A closely related approach in cutlery is based on laminated tool steels. Lamination combines complex layers so that the resulting material can resist both wear and mechanical shocks. In industry, this principle is applied, for example, in drills, milling cutters, or industrial knives, where complex layers provide the cutting edge. At the same time, the more rigid core increases the reliability of the entire tool. In cutlery, we find an analogy in traditional Japanese blades of the “San-mai” type, where hard carbon or martensitic steel is clad with softer and tougher steel. Practical experience confirms that such a construction extends service life and increases edge reliability even under intensive use.

Laminated steels, such as San–Mai constructions [10], combine a hard core steel with two outer layers. This approach offers both economic and functional benefits: it saves expensive material, enhances toughness and resistance to fracture, facilitates repairs, and, when stainless alloys are used, improves corrosion resistance.

A specific group is formed by powder steels [11], produced by the method of powder metallurgy. This process makes it possible to achieve a high carbon content without the cast-iron effect, applies to materials with poor machinability, and ensures a uniform microstructure with finely dispersed carbides. The result is a combination of high strength, wear resistance, and stability during heat treatment.

A comparison of these methods [11,12] demonstrates their complementary benefits: forge welding provides unique aesthetics and traditional character, and laminated steels balance cost and functionality. In contrast, powder steels deliver the highest level of mechanical properties thanks to modern production technology.

Recent studies also highlight the use of rare earth metals and refined casting techniques (e.g., electroslag remelting and twin-roll casting) to further control carbide precipitation and micro segregation. These methods, while primarily used in high-grade tool steels, are directly transferable to advanced cutlery production, where performance optimisation is paramount [3].

Research in the field of laminated and specially alloyed steels shows that combining multiple layers or surface treatments makes it possible to achieve balanced mechanical properties, analogous to the requirements placed on knife blades—namely, high edge hardness while maintaining a rigid core [8]. Functional laminated and multi-layered materials prove that by combining different metallic layers, materials with synergistic properties can be obtained, similar to composite materials [9,10,11,12]. For example, the lamination of bronze and stainless steel (SS316L) results in high strength (876 MPa), good toughness, and, at the same time, oxidation resistance. This approach is directly transferable to cutlery as a modern equivalent of historical Damascus blades, where different layers provide the blade with a combination of a hard edge and a rigid core [13,14].

Advanced Mn steels utilising TRIP (Transformation Induced Plasticity) and TWIP (Twinning Induced Plasticity) effects achieve an exceptional combination of strength and ductility exceeding 40%. The mechanism is based on microstructural changes, where deformation triggers martensitic transformation and twinning, ensuring toughness at high hardness [15]. In cutlery, this principle can serve as inspiration for designing steels capable of withstanding high loads without brittle fractures, which are typical for combat or outdoor knives. Similarly, research on martensitic steels for nuclear applications shows that a suitable choice of alloying elements (e.g., the addition of W or V) can improve toughness. At the same time, excessive Cr and Mn may lead to higher brittleness [16]. These findings can be applied to the development of alloyed blades with optimised properties.

Overall, it can be stated that current scientific work in the field of laminated and surface-treated steels points the way toward the production of a modern “Damascus,” which could combine historical layering techniques with the most advanced methods of materials engineering. For cutlery, this means the possibility of designing blades with even greater resistance, edge hardness, and controlled toughness, thereby advancing traditional knife-making principles to the level of modern science and technology.

The research aims to analyse and compare the properties of selected special steels used in the cutlery industry and to assess their potential in terms of functional and aesthetic parameters. The intention is to identify key factors influencing blade quality and to formulate conclusions that enable the optimisation of material selection and technological processes for specific applications. A significant contribution lies in the fact that this issue has not been sufficiently elaborated in the current scientific literature; the results, therefore, provide new insights and fill a gap in published studies.

Materials research in the field of special knife steels has so far relied mainly on the experience of manufacturers, who, however, do not possess sufficiently sophisticated methods for detailed characterisation of materials. The present study, therefore, focuses on the systematic analysis of three groups of special steels used in cutlery: Damascus, laminated, and powder-metallurgical. It brings new knowledge to the field of materials engineering of special knife steels, expands the existing scientific literature, and provides practical guidance for the targeted use of these materials in cutlery as well as in broader technical applications.

## 2. Materials and Methods

For the experiment and the production of Damascus knives, three different semi-finished steel products were selected: VG10 (with stainless steel edges laminated with nickel and a VG10 steel core; full name VG10 Stainless/Nickel Damascus DPS San Mai–31 layer; manufacturer: TAKEFU SPECIAL STEEL CO., LTD., 21-2-1, Shiromaru-cho, Echizen-city, Fukui 915-0857 Japan; no equivalent designation), the powder-metallurgical damasteel RWL_34_^TM^ (manufacturer: Damasteel^®^ AB, Stallgatan 9, SE-815 76 Söderfors, Sweden; no equivalent designation), and laminated steel composed of K110 and N695 (K110 as the core steel, manufacturer: BÖHLER Edelstahl GmbH & Co KG, Mariazeller-Straße 25, 8605 Kapfenberg, Austria; K110 steel equivalent designation mat. No. 1.2379; X153CrMoV12; AISI D2; ČSN 19 573 and N695 steel equivalent designation mat. No. 1.4125; X105CrMo17; AISI 440C; ČSN 17 241) from three different producers. In addition, one sample of layered Damascus steel of our own manufacture was prepared, consisting of commercially available steels K720 and K600 (BÖHLER Edelstahl GmbH & Co KG; K720 steel equivalent designation mat. No. 1.2842; 90MnCrV8; AISI O2; ČSN 19 312 and K600 steel equivalent designation mat. No. 1.2767; 45NiCrMo16; no other equivalents). The experimental materials were supplied as semi-finished steels. VG10, RWL_34_^TM^, and K600+K720 were delivered in the annealed condition and subsequently manufactured into blades and heat-treated in our laboratory. The laminated steel K110+N695 was supplied in the quenched and tempered state by the producer. All analyses were therefore conducted on heat-treated samples representing the final knife blade condition.

The chemical composition of the steels was measured using an optical emission spectrometer Q4 TASMAN (BRUKER GmbH, Dynamostraße 19, 68165 Mannheim, Germany). Microstructural investigation of the steels was carried out using a confocal laser microscope Olympus LEXT OLS 5000 (Olympus Corporation, Ishikawa-machi, Hachioji-shi, Tokyo, Japan) and a scanning electron microscope TESCAN VEGA 3 XMU equipped with an Oxford 80 EDS (Energy Dispersive Spectroscopy) analyzer (Oxford Instruments plc, Abingdon OX13 5QX, Oxfordshire, England), as well as a TESCAN VEGA 3 (TESCAN ORSAY HOLDING, a.s., Libušina třída 21, 623 00 Brno-Kohoutovice, Czech Republic) equipped with a Bruker XFlash EDS analyser (Bruker Corporation, 40 Manning Road, Manning Park Billerica, MA 01821 USA). (TESCAN ORSAY HOLDING, a.s., Libušina třída 21, 623 00 Brno-Kohoutovice, Czech Republic).

After heat treatment, the hardness of the knives was measured according to Rockwell, following the ČSN EN ISO 6508-1 standard (ISO 6508-1:2023), using an ERNST AT 250X hardness tester (CISAM-ERNST s.r.l., Induno Olona, VA, Italy). The measurements were performed under a load of 150 kgf for 10 s, using a diamond cone indenter (Rockwell C scale, HRC). Within the experiment, microhardness of the basic structural components of the steels was also measured using a Hardness Tester HM-200 (MITUTOYO CORPORATION, Sakado, Takatsu-Ku, Kawasaki, Kanagawa, Japan), with a test load of 0.05 HV and a dwell time of 10 s, according to the ČSN EN ISO 6507-1 standard (ISO 6507-1:2023).

The input material for the production of Damascus knives made of VG10, with stainless steel edges laminated with nickel and a VG10 steel core, was manufactured by a technology involving several processing steps, such as welding of individual layers, hot forming, annealing, and cold forming. The second experimental material, RWL_34_^TM^, was produced by powder metallurgy. The third material sample was laminated steel composed of K110 and N695 with a nickel interlayer (supplied by the manufacturer with recommended heat treatment: hardening—step heating to an austenitizing temperature of 1020–1070 °C, holding for 15–30 min, followed by oil quenching; tempering—double tempering with a holding time of 2 h at 520 °C, with the final tempering 30–50 °C below the previous tempering temperature to achieve secondary hardness, followed by slow cooling in the furnace). For the fourth sample, the input materials were steels K600 and K720, supplied in the annealed soft condition for further processing.

### Technology of the Knife Material Production

Our research employed two significant methods for producing Damascus knife blades, each chosen based on the input material. The first method involved blade grinding from a steel plate, using experimental materials such as VG10, RWL_34_^TM^, and laminated K110+N695. The second method, using experimental material K600+K720, was forge welding.

**Figure 1:**
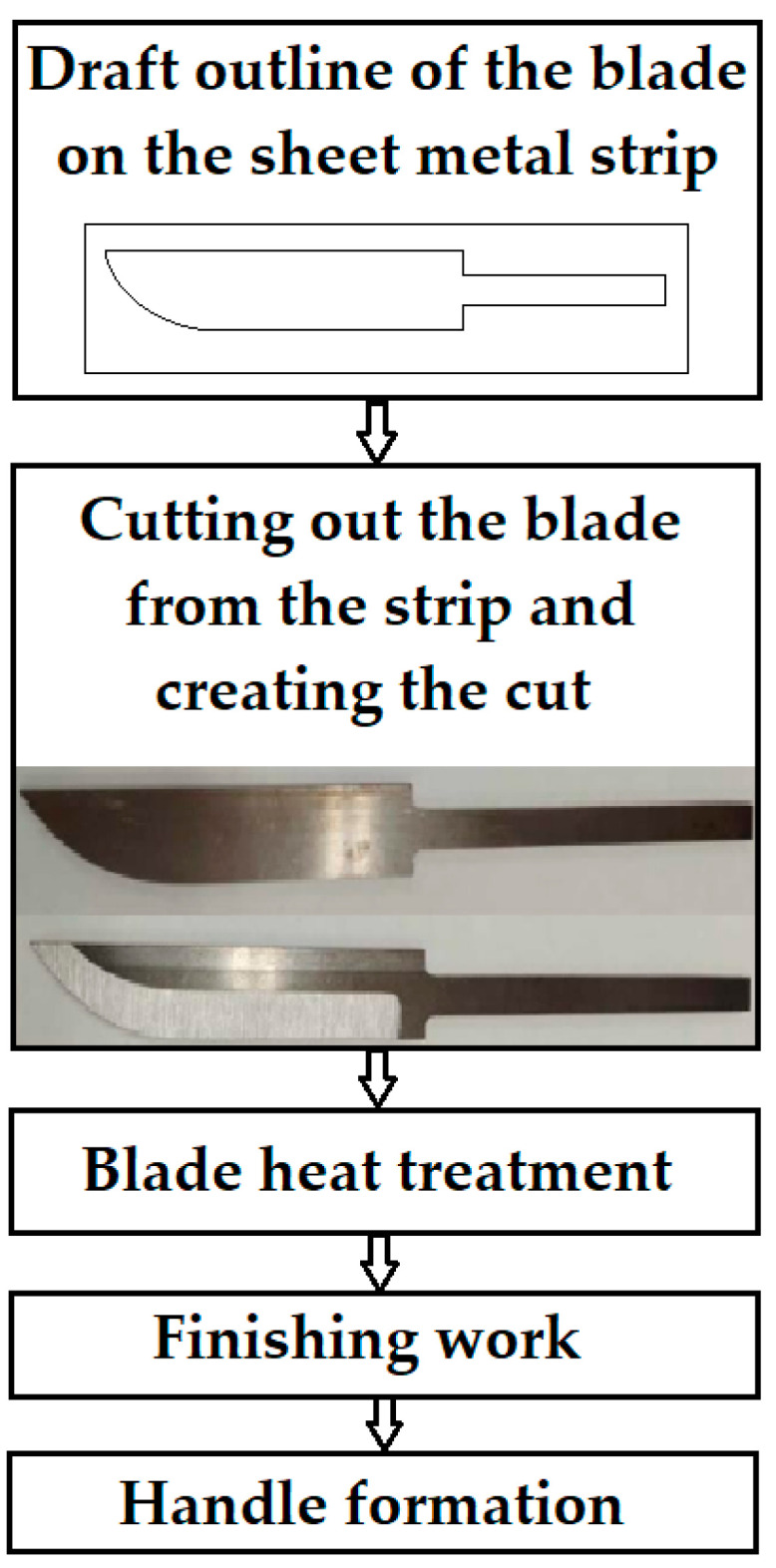
Knife manufacturing process by grinding.

Blade grinding, a straightforward process, involves the direct manufacture of a knife blade from a steel plate. This method, one of the simplest in production, is based on a steel strip that remains unaltered during production. The process can be broken down into several steps, as illustrated in Figure 1. Using this method, we produced samples from VG10, RWL_34_^TM^, and laminated K110+N695.

**Figure 2:**
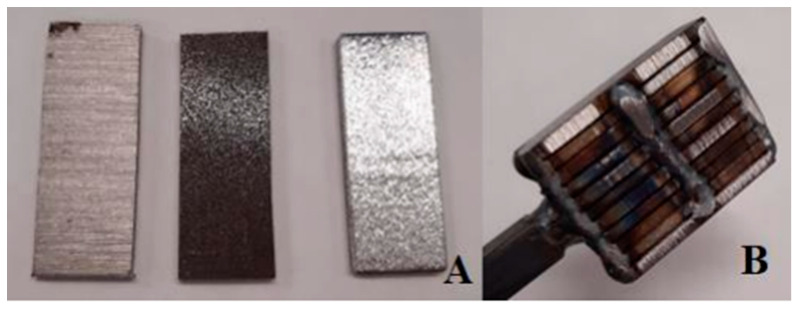
Process of Damascus steel production by forge welding; (**A**)—input materials, (**B**)—press anvil before forge welding.

For the final sample made of K600 and K720 steels, we opted for the meticulous forge welding process. This technology involves the careful cutting and surface preparation of the steel, as shown in Figure 2. The steel is cut to dimensions smaller than those of the hammer or press anvil (Figure 2A), and the surface is roughened to ensure better bonding of the individual plates due to surface irregularities. The steel is then alternately stacked and welded into a so-called billet (Figure 2B). The billet is heated to approximately 850 °C with the application of flux, specifically borax (sodium tetraborate). This is followed by reheating to approximately 1300 °C, with sufficient holding time to ensure uniform heating. The individual layers are then joined by precise hammer blows on the anvil. After the initial welding, the billet is returned to the forge and worked into the required shape. To refine the final pattern of the blade steel, the material is repeatedly folded, which increases the number of layers. Finally, the shape of the knife is cut from the Damascus steel thus produced, followed by the steps shown in Figure 1.

The heat treatment of the samples was carried out under different regimes depending on the type of steel:

The knife made of VG10 steel (with stainless steel edges laminated with nickel and a VG10 steel core) was quenched and tempered. Before the process, the steel was wrapped in a single layer of quenching foil with a thickness of 0.05 mm. The heat treatment regime included step heating to the austenitizing temperature, where the first heating was to 850 °C with a holding time of 30 min, followed by heating to the austenitizing temperature of 1060 °C and quenching in oil. The steel was then tempered at 175 °C for two hours with slow air cooling. The heat treatment resulted in a hardness of 52 ± 1 HRC.

**Figure 3:**
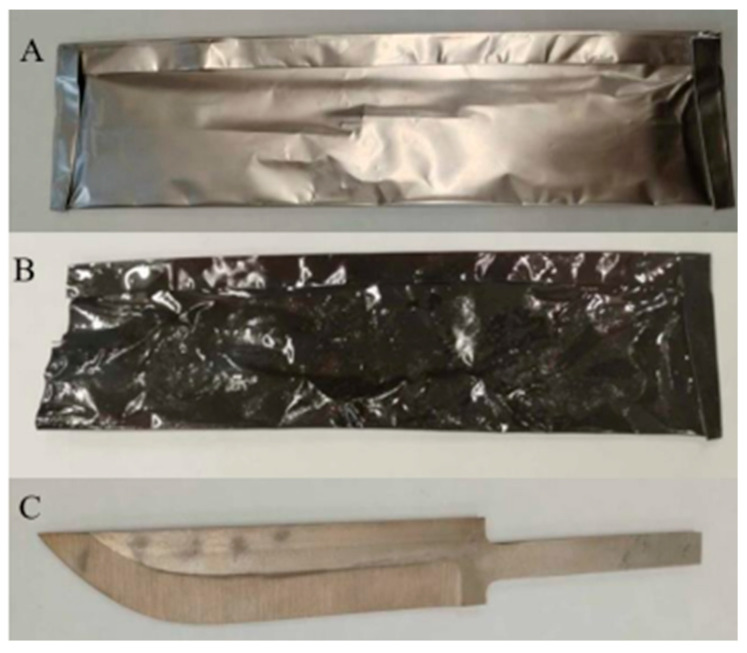
Quenching of the knife made of RWL_34_^TM^ steel in protective hardening foil; (**A**)—hardening foil before quenching, (**B**)—after quenching, (**C**)—knife blade protected against oxidation during heat treatment.

The knife, made of RWL_34_^TM^ steel (produced by powder metallurgy), was heat-treated through quenching and tempering. Before heating to the austenitizing temperature, the steel was wrapped in a single layer of quenching foil, see Figure 3. The steel was heated to the austenitizing temperature of 1060 °C and quenched in oil. This was followed by tempering at 175 °C for 2 h with slow air cooling. A blade hardness of 58 ± 1 HRC was achieved.

The third knife sample was made of laminated steel composed of K110 (core steel) and N695 with a nickel interlayer. The manufacturer supplied this steel in the quenched and tempered condition. The core steel K110 reached a hardness of 61 ± 1 HRC.

The knife made of Damascus steel K600 and K720, produced by forge welding, was heat-treated in a similar way to the VG10 and RWL_34_^TM^ steels. Quenching was carried out at 820–840 °C. Before quenching, the knife was wrapped in two layers of quenching foil to protect it against oxidation. After hardening, the blade was quenched in oil. Tempering was performed at 200–230 °C for 2 h with air cooling. The final hardness of the knife (HRC) was not determined due to the different harnesses of the individual layers. To evaluate the hardness of the individual layers, Vickers microhardness measurements were carried out on prepared metallographic specimens.

## 3. Results

### 3.1. Chemical Composition of the Experimental Materials

The chemical composition provided by the steel suppliers was compared with the measured chemical composition. The chemical composition of the input materials is essential information for the subsequent heat treatment of the manufactured knife blades and for the later interpretation of the evaluated microstructures. The chemical composition was measured six times on each sample. Sample 4 was measured after processing, i.e., after forge welding, as the final material for knife production.

As shown in Table 1, variability in the content of certain elements is present. The structure/build of the steel itself causes this. When measured from the sample surface, only the outermost layer was analysed, and not the overall chemical composition of the material. For these specific types of steels, it was not possible to determine the total composition using the standard OES (Optical Emission Spectroscopy) method. This analysis was therefore complemented by chemical composition measurements using EDS (Energy Dispersive Spectroscopy), where individual structural components of the steels were measured separately on metallographic specimens. From the viewpoint of chemical composition, the materials are inherently non-homogeneous, a characteristic that we anticipated and are managing effectively.

The comparison of the declared and measured chemical composition of the experimental materials revealed certain deviations in the content of some alloying elements. These differences can be attributed primarily to the heterogeneous structure of multi-layered and laminated steels, where optical emission spectroscopy measurements taken from the sample surface capture only the composition of a specific layer rather than the entire material volume.

This limitation of the standard OES method in accurately representing the average composition of multi-layered composites, as seen in Table 1, underscores the need for supplementary analysis [17]. The results were therefore supplemented by SEM (Scanning Electron Microscopy)/ EDS analysis on metallographic specimens, which enabled the determination of the composition of individual structural components separately.

### 3.2. Microstructural Analysis

Microstructural analysis was carried out on metallographic specimens of all knife samples after heat treatment. The examined specimens were cut from raw material stock and subsequently heat treated in laboratory furnaces according to the recommended regimes for each steel grade. The microstructural results presented therefore correspond to the heat-treated state of the steels, not to finished knife products. This preparation allowed observation of the core material, interlayers (nickel or forge weld zones), and outer layers. Representative samples of the steels were taken and sectioned using a precision metallographic saw, Secotom-60 (Struers ApS, Ballerup, Denmark). The samples (4 pieces) were mounted in round polypropylene moulds Ø 25 mm and 27 mm high, using transparent metallographic UV-curable resin QPREP KEM 50 UV, and cured for 1 min in a QMOUNT device (QATM, Mammelzen, Germany). The specimens were prepared using standard procedures on a single-disk grinding and polishing machine QPOL 250 M1 (QATM, Mammelzen, Germany).

The metallographic preparation procedure included grinding (QATM abrasive paper Ø 250 mm SiC F180, F320, F800, F1200, F2500, and F4000, wet grinding with cold water). After the final grinding step, the samples were polished in three stages (BETA polishing cloth with Dia-Coplete Poly 9 µm suspension, GAMMA polishing cloth with Dia-Coplete Poly 3 µm suspension, and OMEGA polishing cloth with Eposal 0.06 µm suspension).

The microstructure of the samples was observed in both unetched and etched conditions. Since no universal etchant exists for these heterogeneous steels, different reagents and etching times were required to reveal the individual structural layers reproducibly. The etchants and conditions applied are summarised in Table 2. This limitation aligns with established metallographic practice, where it is widely recognised that no single etchant is suitable for simultaneously revealing all structural features in multi-phase or compositionally heterogeneous steels. The selection of etching reagent must therefore be tailored to the specific phases present, and often requires a compromise between under- and over-etching of adjacent layers [18].

The prepared specimens, i.e., the microstructures of individual samples, were observed using both light optical microscopy and SEM, followed by evaluation of the chemical composition of structural components using EDS.

**Figure 4:**
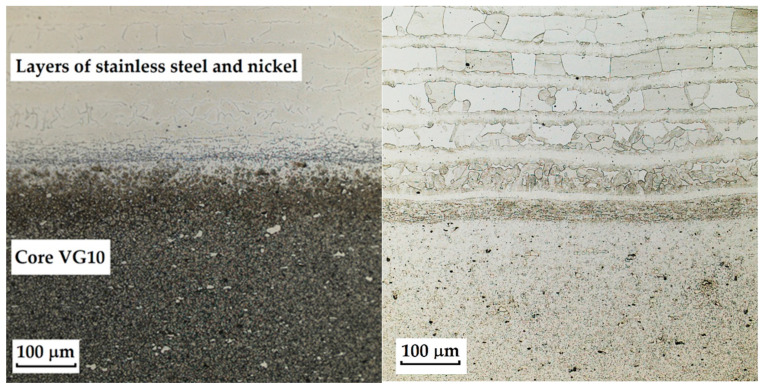
Microstructure of a VG10 steel sample with edges laminated with nickel-plated stainless steel and a VG10 steel core; on the left, a poorly etched Kroll structure with etched core steel and visible carbides; on the right, a well etched V2A structure with clearly visible lamination of stainless steel and VG10 core steel and visible carbides.

For VG10 steel (with stainless steel edges laminated with nickel and a VG10 core), the most suitable etchant was V2A at 20 °C. This etchant is primarily intended for austenitic steels when used at approximately 60 °C. It consists of nitric acid (HNO_3_), hydrochloric acid (HCl), and water (H_2_O) (QATM, Mammelzen, Germany). Other etchants (Kroll: nitric acid (HNO_3_), hydrofluoric acid (HF), and water (H_2_O); Nital 3%: nitric acid (HNO_3_) and ethanol (C_2_H_5_OH)) did not yield satisfactory results. With Kroll’s reagent, the core steel was overetched, and only carbides were observed. Nital 3% produced no result, as the structure did not etch. The microstructure etched with Kroll’s reagent and V2A is documented in Figure 4.

**Figure 5:**
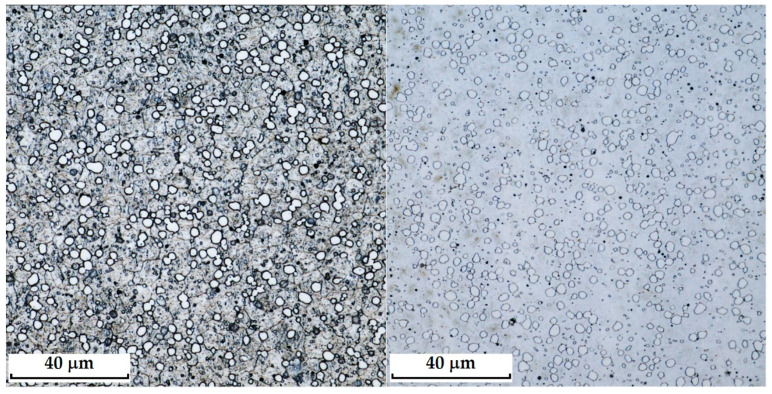
Microstructure of a sample of RWL_34_TM steel produced by powder metallurgy technology; on the **left**, etched Kroll structure with observable carbides and original grain boundaries; on the **right**, etched V2A structure with visible carbides.

RWL_34_^TM^ steel (produced by powder metallurgy) responded best to Kroll’s reagent and V2A, where carbides were most pronounced in the etched structure. The microstructure after etching is shown in Figure 5. With Kroll’s reagent, the original grain boundaries were also observed, whereas with V2A, they completely disappeared, leaving only carbides visible in the microstructure. Both etchants, however, can be considered suitable.

**Figure 6:**
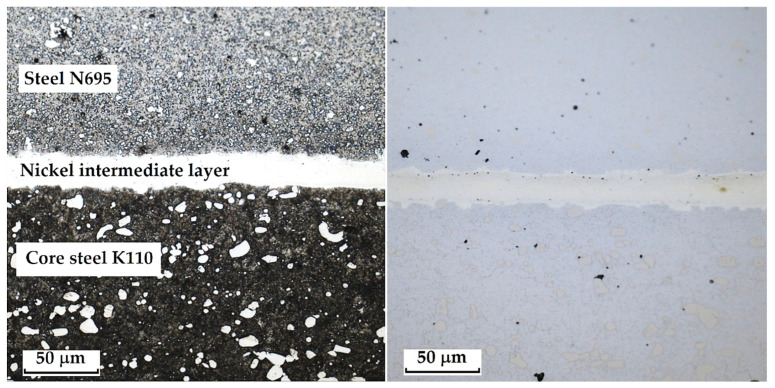
Microstructure of a sample of K110 steel (core steel) and N695 steel with a nickel interlayer; on the **left**, the etched Kroll structure with visible carbides in both steels, the etched core steel and the nickel interlayer; on the **right**, the etched V2A structure with visible carbides and original grain boundaries in the core steel, the nickel interlayer, but not completely etched N695 steel layer.

The laminated steel composed of K110 (core steel) and N695 with a nickel interlayer produced the best results when etched with 3% Nital for 4 min. When Kroll’s reagent was used, the microstructure of the core steel was overetched, but carbides in both structures, as well as the nickel interlayer, were clearly visible. With 3% Nital, the microstructures were not as strongly overetched; the original grain boundaries, carbides, and the nickel interlayer were distinguishable, although the microstructure of N695 steel was more clearly observable when etched with Kroll’s reagent (see Figure 6). It follows that for a comprehensive observation of this laminated steel, both etching methods may be used, each allowing better visibility of specific layers that respond more favourably to the given reagent.

**Figure 7:**
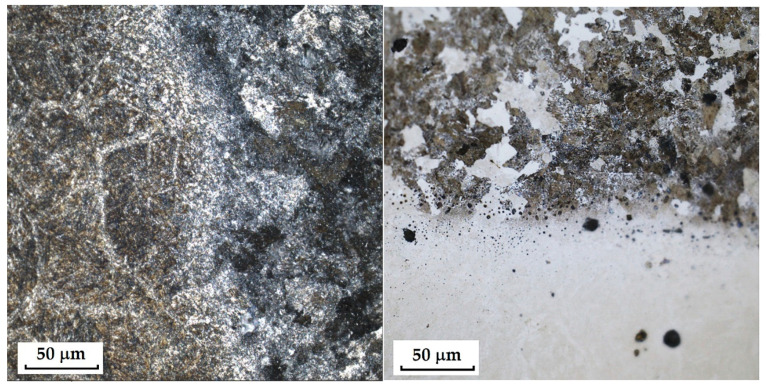
Microstructure of a sample of forge-welded Damascus steel K600 and K720; on the **left**, an over-etched Kroll structure with observable boundaries of the original austenitic grains, a transition forge weld between the steels; on the **right**, an etched Nital 3% structure with visible microstructures of both steels, a transition area between the steels.

The last sample of forge-welded Damascus steel K600 and K720 showed the best results in microstructural observation when etched with 3% Nital. Other etching methods proved too aggressive for this type of steel. When etched with Kroll’s reagent, the microstructure was strongly overetched; however, it was possible to observe the boundaries of the original austenitic grains in one of the steels (K600) as well as the transition zone of the forge weld. Figure 7 documents both etching methods, Kroll and 3% Nital.

The results of the etching tests demonstrated that, due to the different chemical and phase compositions of the materials used, it was not possible to apply a universal etchant for all steel types. VG10 steel showed the best contrast when etched with V2A at 20 °C, while Kroll’s reagent caused overetching of the core layer and highlighted only carbides. RWL_34_^TM^ steel responded well to both Kroll and V2A; the former enabled the observation of original grain boundaries, while the latter primarily highlighted carbides. The laminated steel K110+N695 achieved the best results with 3% Nital after a longer exposure time. However, for comprehensive characterisation, it was necessary to combine Nital and Kroll, as each etchant emphasised a different layer. The forge-welded steel K600+K720 showed the best results when etched with 3% Nital, whereas Kroll was too aggressive for this material.

#### 3.2.1. Light Optical Microscopy Microstructural Evaluation

After determining suitable etchants for revealing the microstructure of all steel samples, microscopic analysis of all microstructural components of the knife materials was performed. The focus was placed on both the individual steel layers and the transition zones, as well as on possible defects, which are documented in Section 3.4, ‘Material Defects and Structural Inhomogeneities’. The results of the microstructural analysis, whether obtained by light optical microscopy or SEM, were further used for Vickers microhardness measurements, since this analysis clearly identified the microstructures from which each knife material was composed.

**1**. **VG10 Steel with Nickel-Plated Stainless Steel Laminated Edges and VG10 Steel Core**

This sample, used for the production of a Damascus knife, is pre-laminated from a 15-layer plated stainless steel VG10. The central layer (core) of VG10 steel is surrounded by alternating layers of stainless steel and nickel, forming a total of 31 layers. Nickel is most often added to block carbon migration in steel. Since this steel has a core of premium VG10 and laminated edges also made of stainless steel, the DPS (nickel layer) would not be necessary from the perspective of carbon migration. However, the manufacturer applied it to enhance the contrast between individual layers and to create an aesthetic separation of the blade zones.

**Figure 8:**
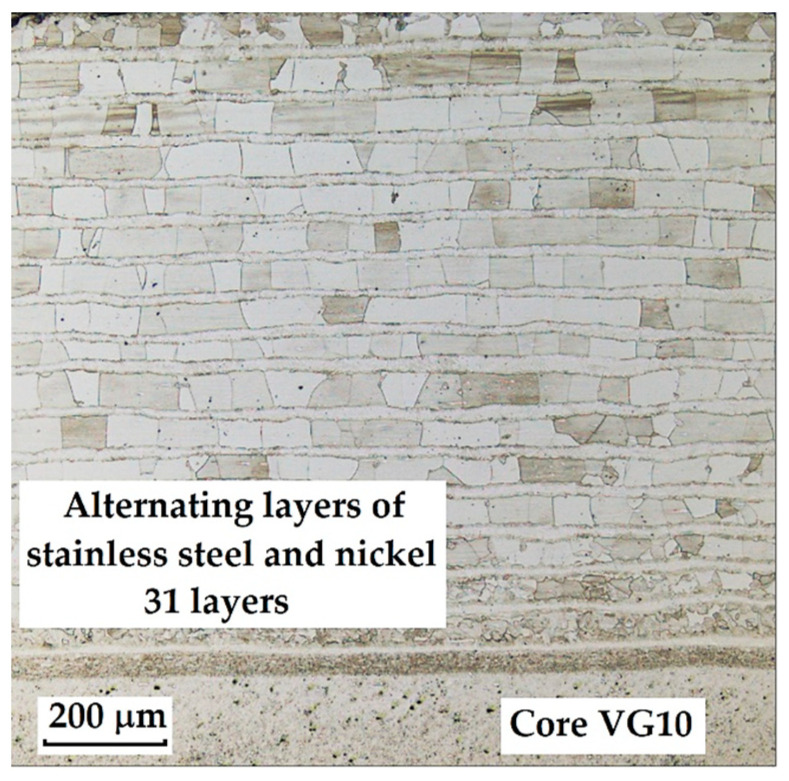
Microstructure of a VG10 steel sample with edges laminated with nickel-plated stainless steel and a VG10 steel core.

**Figure 9:**
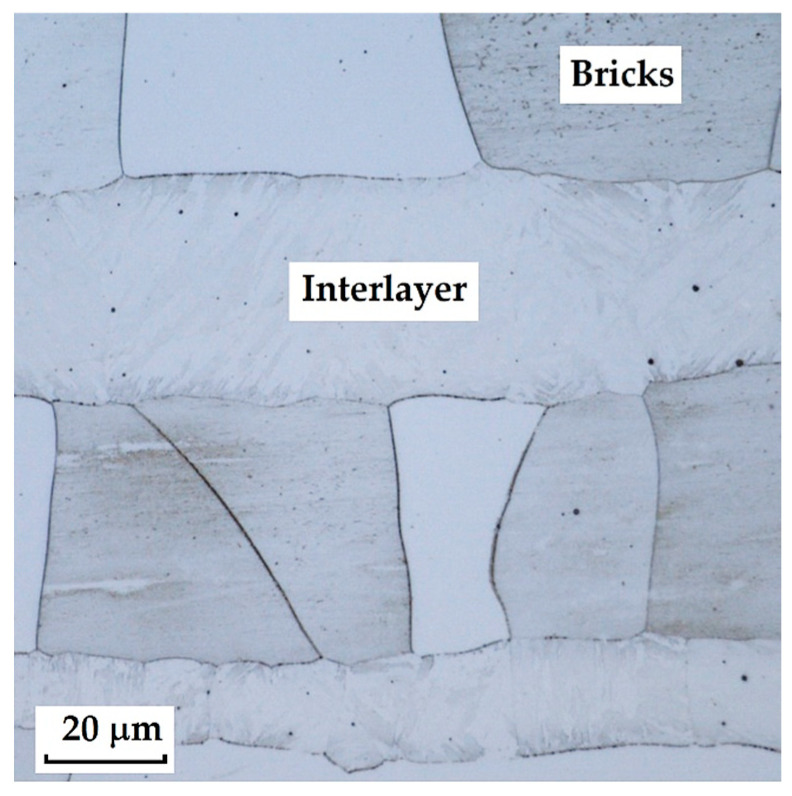
Detail of the microstructure of alternating layers of stainless steel and an interlayer containing nickel.

**Figure 10:**
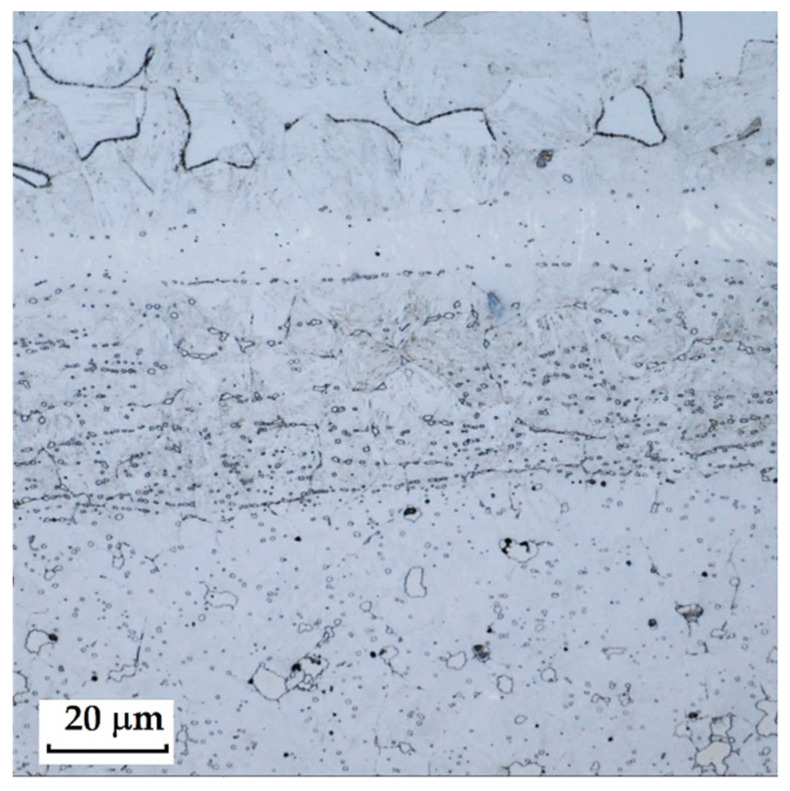
Detail of the microstructure of the transition between the laminated structure and the core.

**Figure 11:**
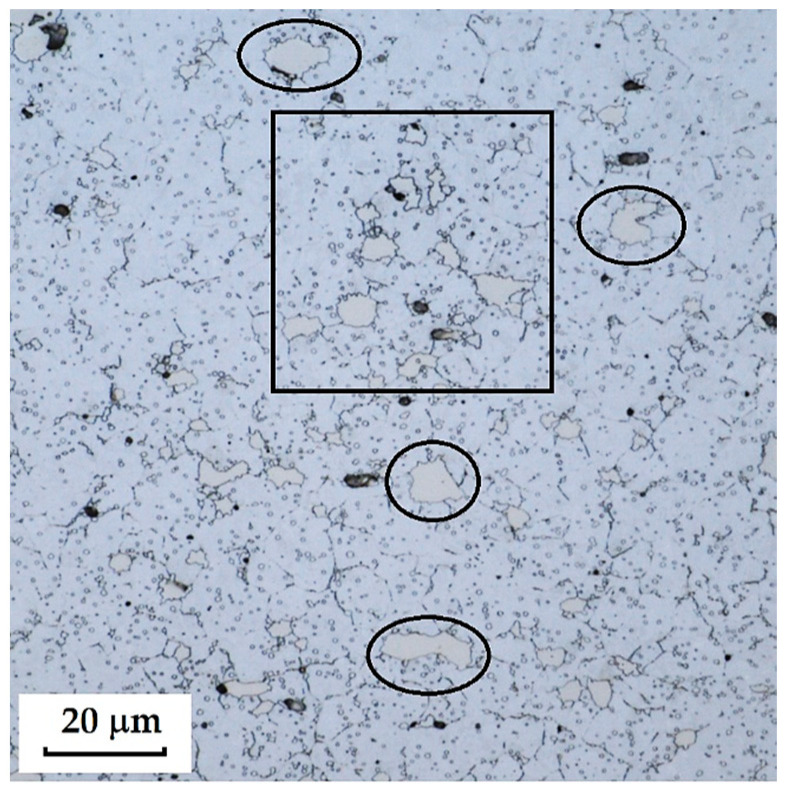
Detail of the core microstructure with precipitated large carbides of various sizes and irregular shapes.

The following images present a crucial aspect of our research-the microstructure of the steel sample. The stainless-steel layers (Figure 8) are documented as bricks; on the metallographic specimen, they appear as individual “bricks”, which is caused by the crystallographic orientation of these folded layers. Details of the microstructure are shown in Figure 9, Figure 10 and Figure 11.

The microstructure of this steel type after heat treatment is expected to be martensitic (observed mainly in the laminated interlayer), with finely dispersed carbides in the VG10 core steel and in the transition structure. However, the images clearly show that for this particular steel sample, an unsuitable heat treatment technology was chosen, as evidenced by the presence of large precipitated carbides in the VG10 core (Figure 11) as well as carbide precipitation at grain boundaries in the transition layer and even in the laminated stainless steel.

**2**. **RWL_34_^TM^ Steel (Steel Produced by Powder Metallurgy Technology)**

**Figure 12:**
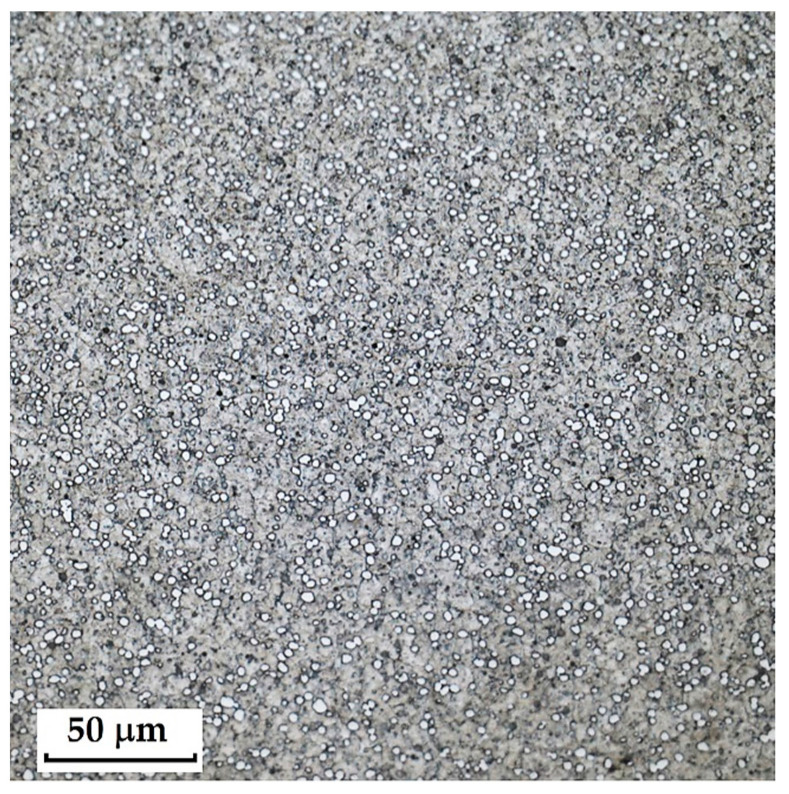
Microstructure of RWL_34_TM steel with fine, evenly dispersed carbides (white particles).

**Figure 13:**
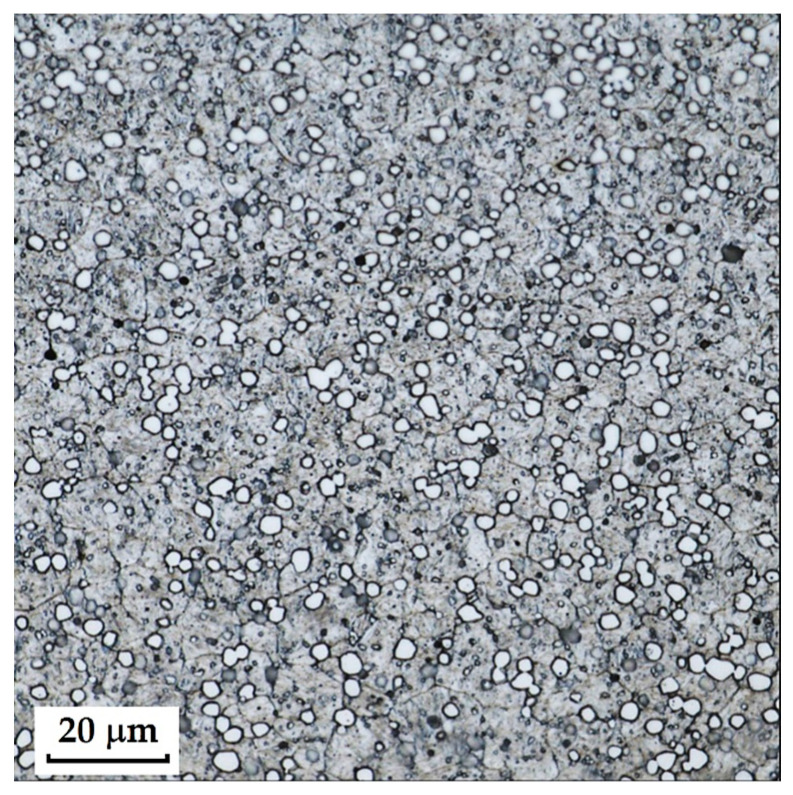
Detail of the steel RWL_34_^TM^ microstructure.

RWL_34_^TM^ steel was produced by powder metallurgy. It is a martensitic stainless Damascus steel suitable for knife production. This steel has a very fine-grained structure, and after heat treatment, it is characterised by fine martensitic needles and finely, uniformly distributed carbides. Figure 12 documents the microstructure of this powder-metallurgy steel. A detailed view of the structure is shown in Figure 13.

**3**. **Laminated Steel Consisting of K110 Steel (Core Steel) and N695 Steel with a Nickel Interlayer**

**Figure 14:**
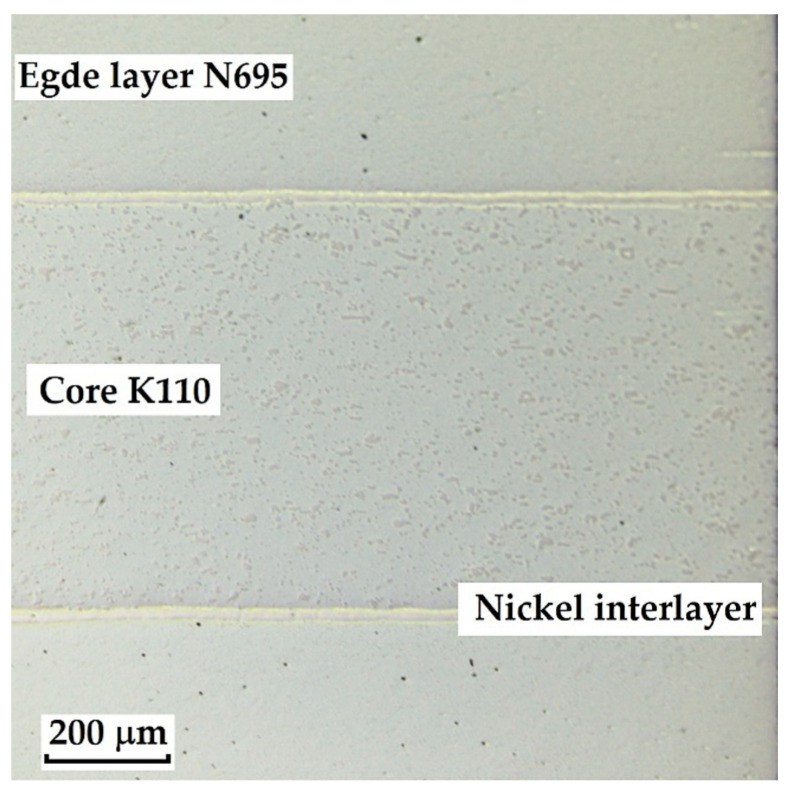
Microstructure of a laminated steel sample consisting of K110 steel (core steel) and N695 steel with a nickel interlayer.

This type of steel represents a combination of knife steel K110 with stainless steel N695. K110 is a high-carbon, ledeburitic chromium tool steel, also known as D2 steel, whereas N695 belongs to the corrosion-resistant stainless-steel category. The structure of this steel consists of a core made of laminated K110 and edges of stainless steel N695. Between the two layers of steel, a thin nickel interlayer (approx. 20 µm) is inserted, as documented in Figure 14.

**Figure 15:**
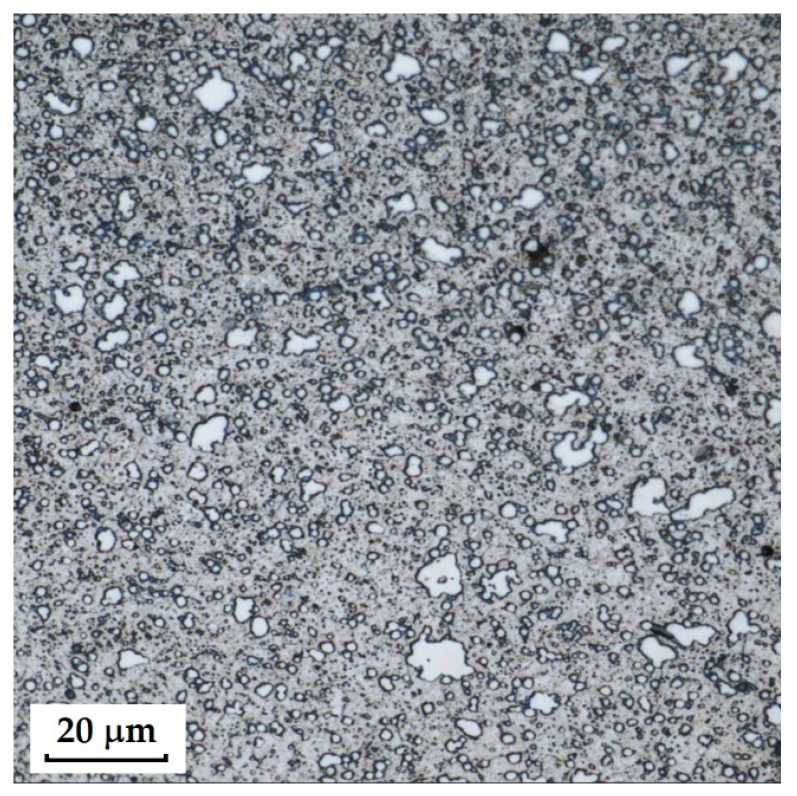
Detail of the microstructure of edge N695 steel with large carbides visible.

**Figure 16:**
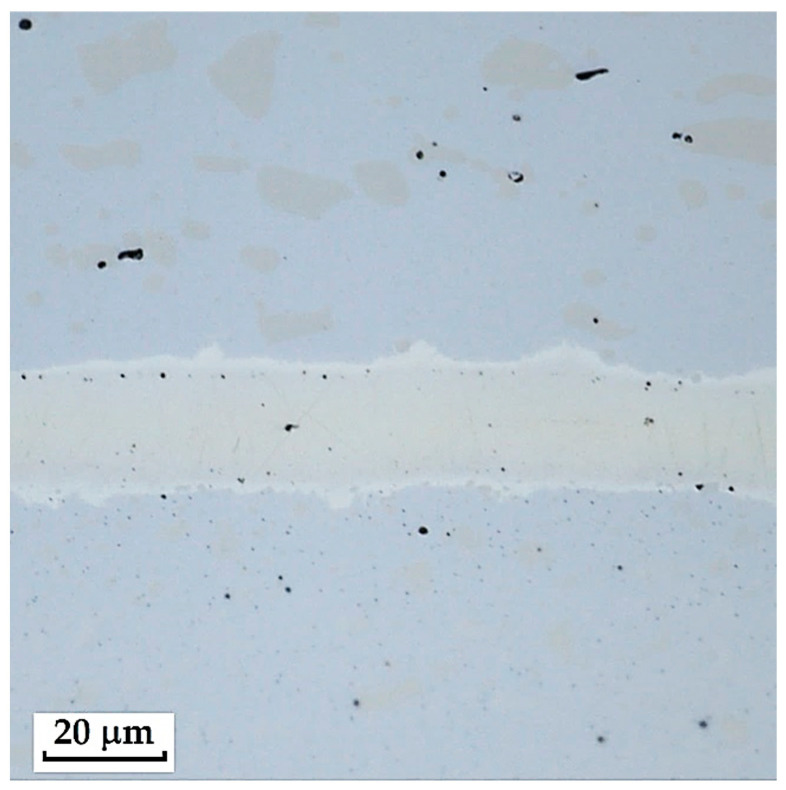
Detail of the microstructure of the nickel interlayer.

**Figure 17:**
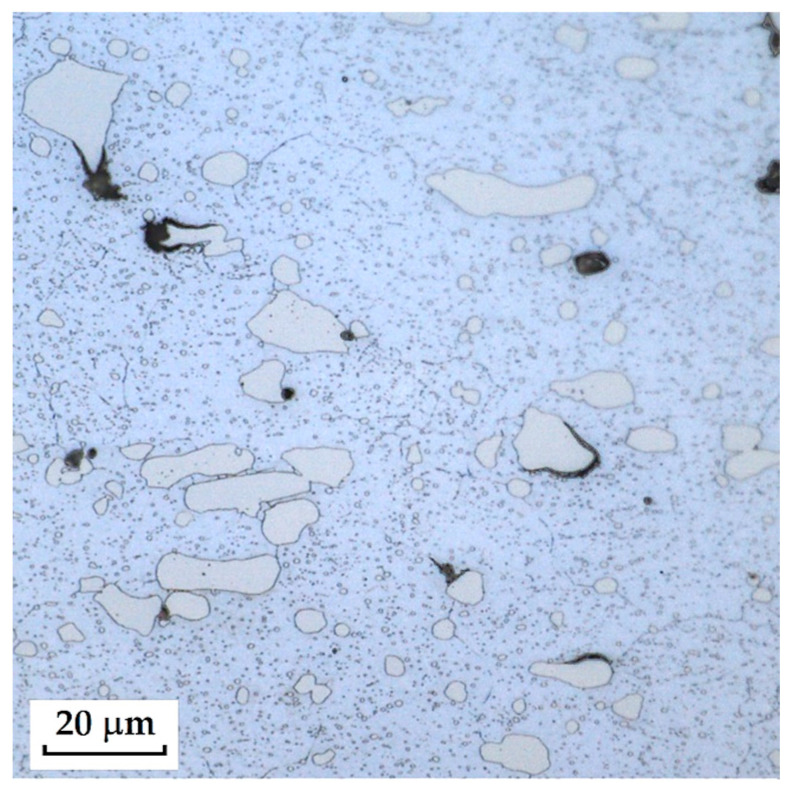
Detail of the microstructure of K110 core steel with large carbides.

This Damascus steel, thanks to its sandwich-like structure, provides the knife produced from it with a design characteristic of Damascus steels, to which the wavy nickel line contributes significantly. Figure 15, Figure 16 and Figure 17 show details of the steel’s microstructure after heat treatment. The microstructural detail in Figure 15 indicates the presence of large primary carbides and fine secondary carbides. Figure 16 documents the detail of the microstructure of the nickel interlayer. Figure 17 documents large carbides in the microstructure of the core steel and the separation of these carbides from the steel matrix.

**4**. **Forge-Welded Damascus Steels K600 and K720**

**Figure 18:**
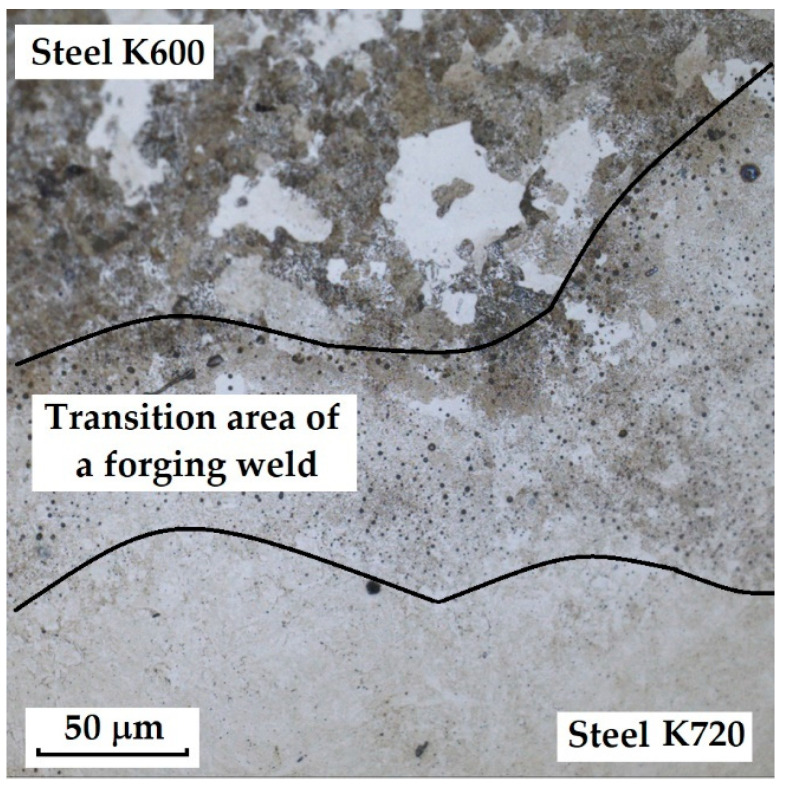
Microstructure of a sample of forge-welded Damascus steel K600 and K720.

**Figure 19:**
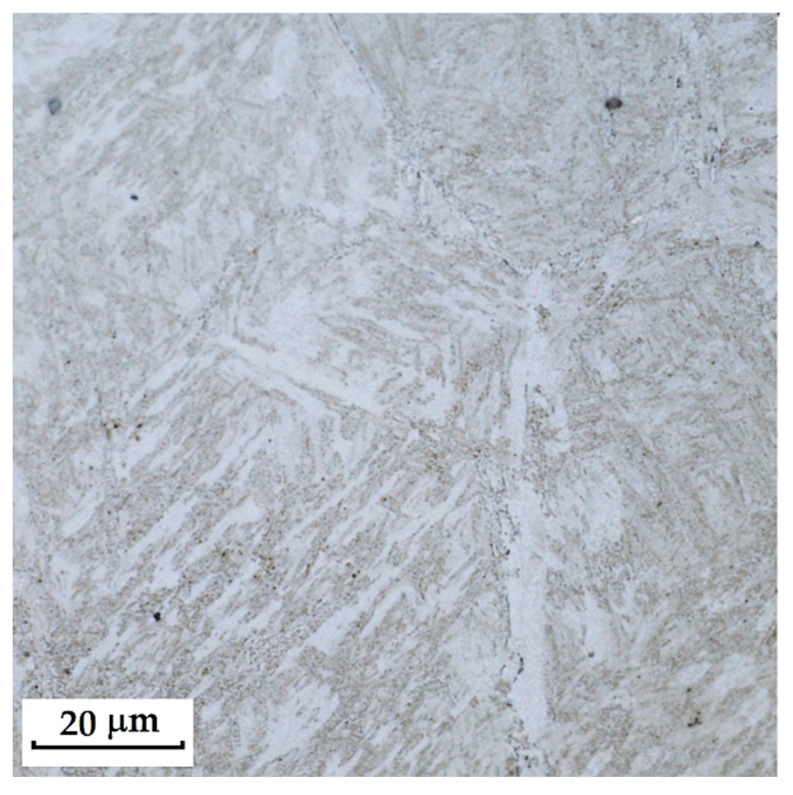
Microstructure of tempered bainite of K700 steel.

**Figure 20:**
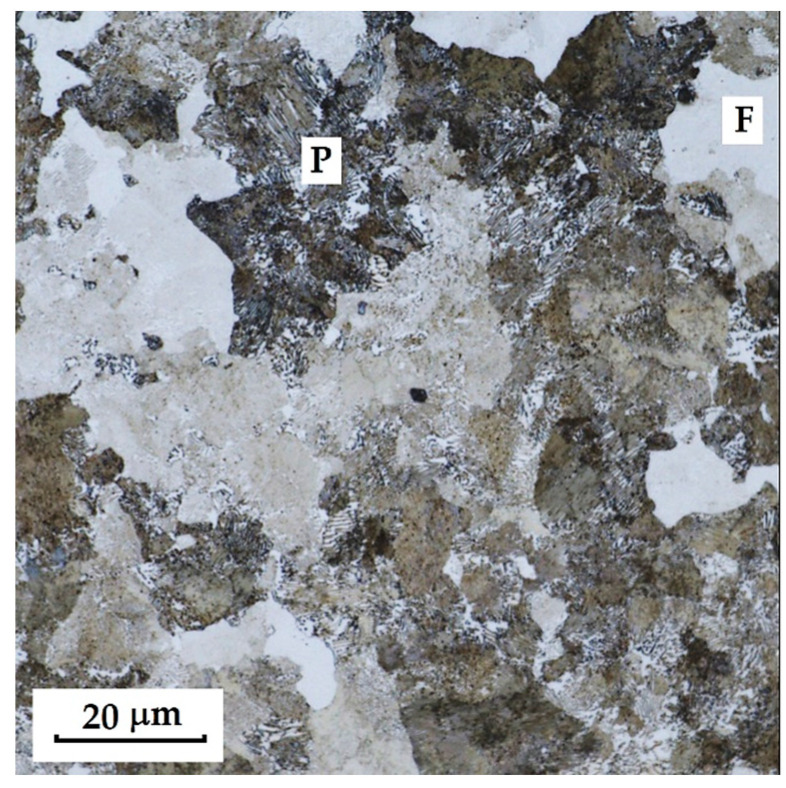
Ferritic-pearlitic microstructure of K600 steel.

This sample differs from the previous ones in its production technology, specifically forge welding. The Damascus steel was produced from K600 and K720 steels. K600 belongs to the group of alloyed tool steels, while K720 is a type of tool steel. Figure 18 documents the microstructure of the sample made from forge-welded Damascus steel. In this sample, three areas with different microstructures can also be observed: the forge weld zone between K600 and K720 steels, the K600 area with a ferritic–pearlitic microstructure (Figure 19; ferrite F; perlite P), and the K720 area, in which a tempered bainitic structure was observed (Figure 20).

#### 3.2.2. SEM and EDS Analysis

For a more detailed study of the microstructure of Damascus steel samples, SEM and EDS analyses were also used. For SEM microstructural evaluation, both the SE (Secondary Electron; TESCAN ORSAY HOLDING, a.s., Libušina třída 21, 623 00 Brno–Kohoutovice, Czech Republic) and BSE (Backscattered Electron; TESCAN ORSAY HOLDING, a.s., Libušina třída 21, 623 00 Brno–Kohoutovice, Czech Republic) detectors were employed at various magnifications. For the assessment of chemical composition, point EDS analysis was primarily used, enabling us to focus directly on specific carbides, interlayers, etc.

**1**. **VG10 Steel with Stainless Steel Edges Laminated with Nickel and a VG10 Steel Core**

**Figure 21:**
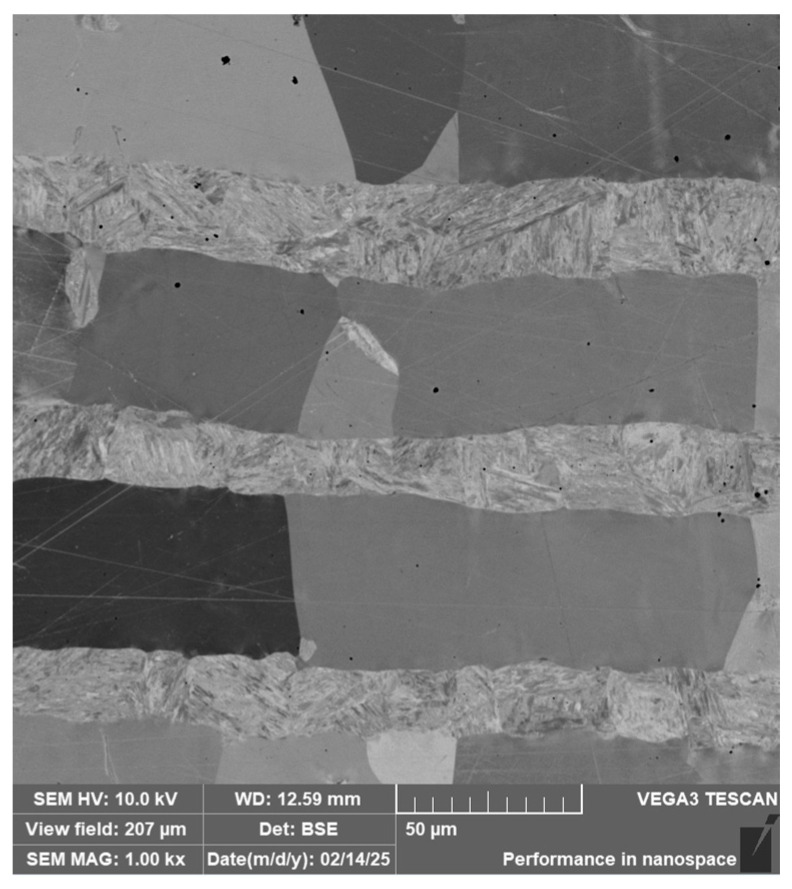
SEM of laminated stainless steel (bricks and interlayer with nickel).

**Figure 22:**
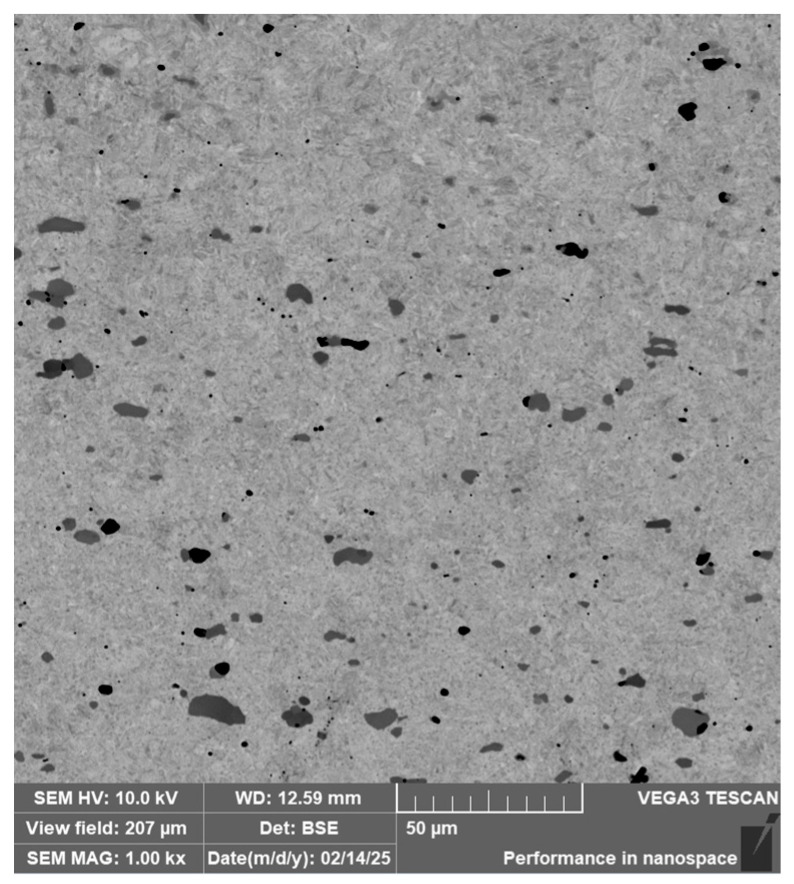
SEM of the VG10 steel core.

**Figure 23:**
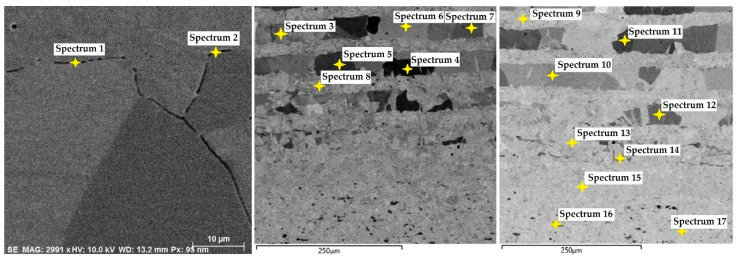
EDS analysis of the VG10 steel sample.

In this steel sample, a martensitic structure was mainly observed in the interlayer between the laminated stainless steels (Figure 21). Figure 22 documents the VG10 core steel with the presence of large, irregularly distributed carbides. The chemical composition of individual structural components was determined by EDS analysis (Figure 23), and the results are presented in Table 3. The table provides the element content in wt. % and the measurement error of ±3σ. EDS analysis identified chromium carbides at grain boundaries, the presence of Ni in the laminated interlayer, and large (Cr,Mo,V)C carbides in the VG10 core steel.

**2**. **Steel RWL_34_^TM^ (Steel Made by Powder Metallurgy Technology)**

**Figure 24:**
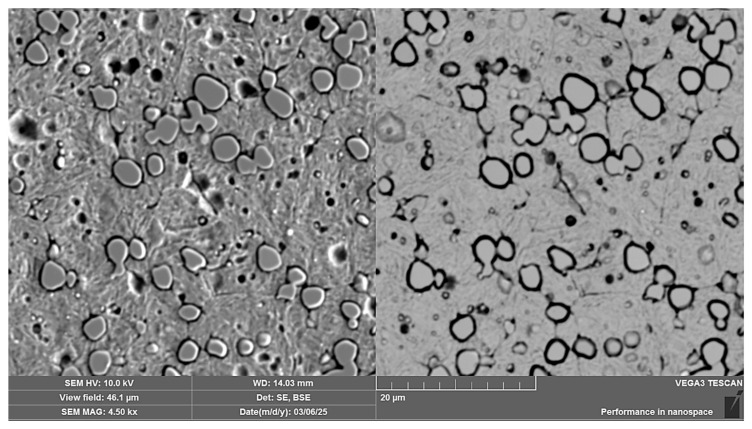
SEM of a sample of RWL_34_^TM^ steel showing fine martensite with small, evenly distributed carbides in the microstructure.

When observing the RWL_34_^TM^ steel sample under SEM, the fine martensitic structure of the matrix and small, uniformly distributed carbides throughout the sample volume were clearly visible. Figure 24 documents the microstructure and carbides of this powder-produced steel. The main advantage of powder metallurgy technology is the absence of structural defects. The image shows sites, i.e., cavities, from which carbides were dislodged during metallographic specimen preparation; therefore, these are not considered defects of the material structure.

**Figure 25:**
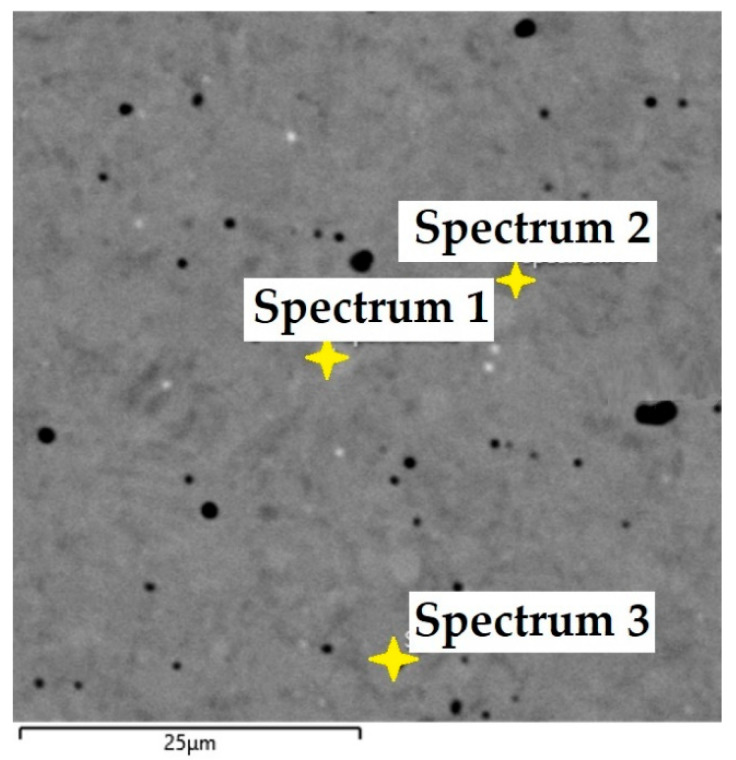
EDS analysis of a steel sample RWL_34_^TM^.

EDS analysis was primarily focused on the identification of carbides, with their chemical composition presented in Table 4. EDS sample RWL_34_^TM^ analysis [wt. %]. and Figure 25. The table provides the element content in wt. % and the measurement error of ±3σ. In this sample, the presence of fine, uniformly distributed (Cr,Mo,V)C carbides in the steel microstructure was confirmed.

**3**. **Laminated Steel Composed of K110 (Core Steel) and N695 with a Nickel Interlayer**

**Figure 26:**
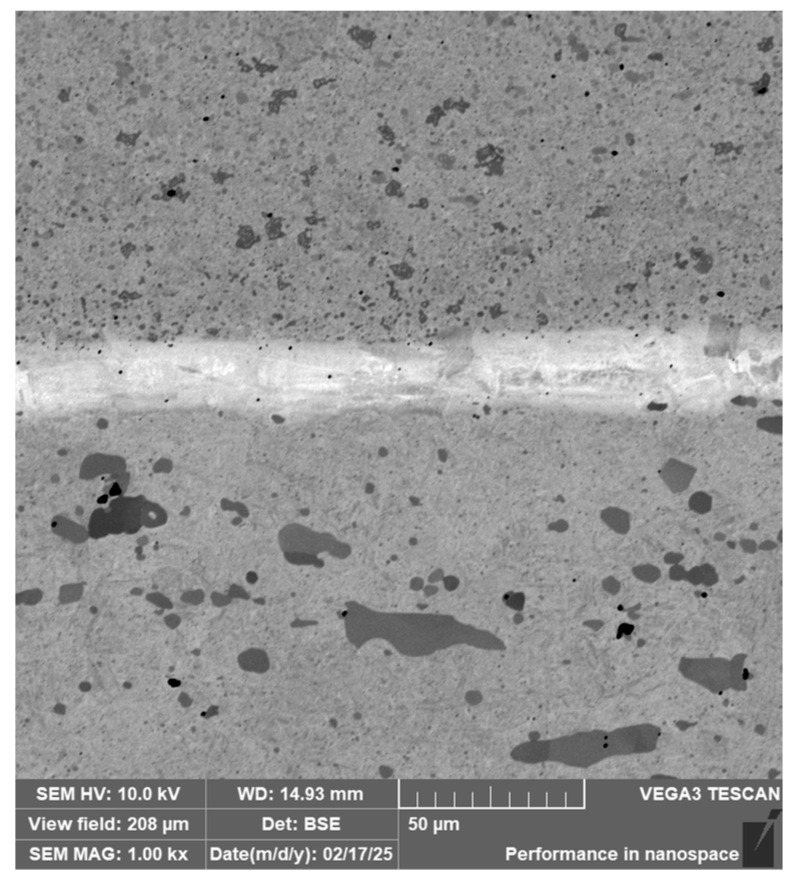
SEM of a laminated steel sample consisting of K110 steel (core steel) and N695 steel with a nickel interlayer.

**Figure 27:**
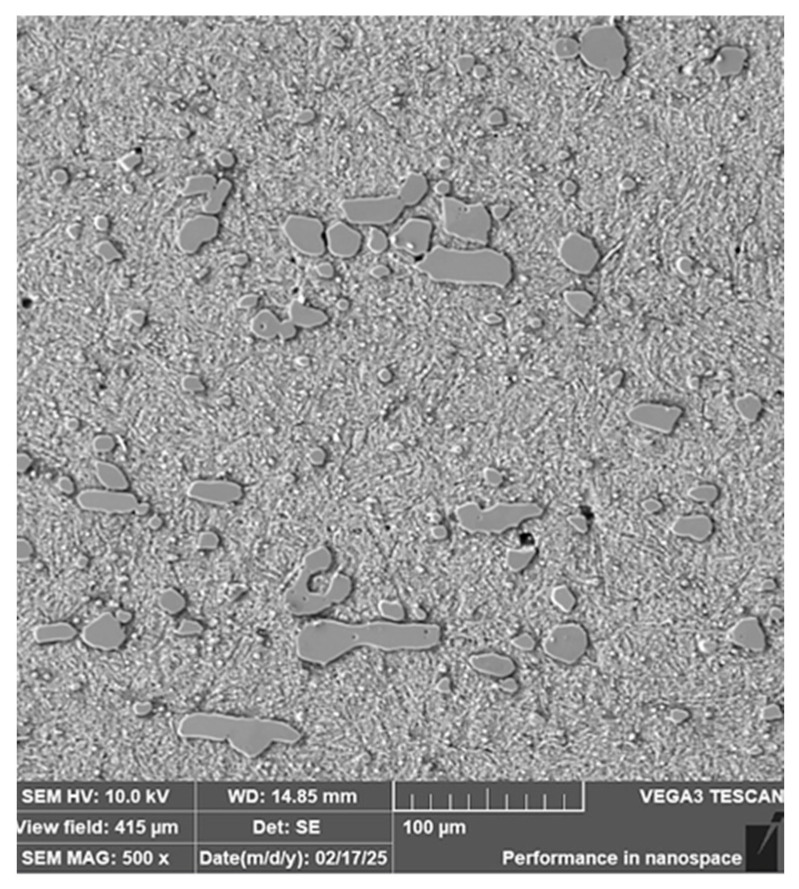
SEM of a K110 steel core sample (core steel) with large carbides.

**Figure 28:**
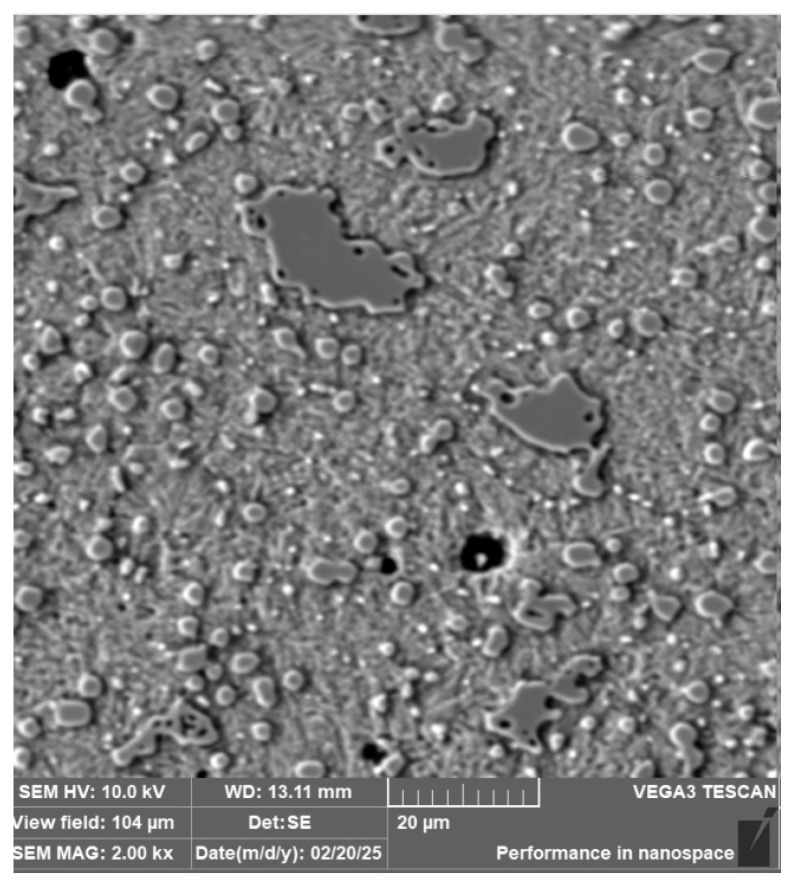
SEM of a sample of edge steel with both fine and large primary carbides.

In this sample, we evaluated three types of structures from which the specimen is composed: laminated K110 steel (the sample core), the edge steel N695, and the nickel interlayer. Figure 26 illustrates the nickel interlayer that separates the two steel types. Figure 27 documents the martensitic microstructure of the core steel with large carbides. In N695 steel, primary (coarser) carbides are observed in the martensitic matrix, along with fine secondary carbides (Figure 28). A clear difference can be seen in the shape and structure of carbides in the microstructures of the core and edge steels, stemming from their different origins.

**Figure 29:**
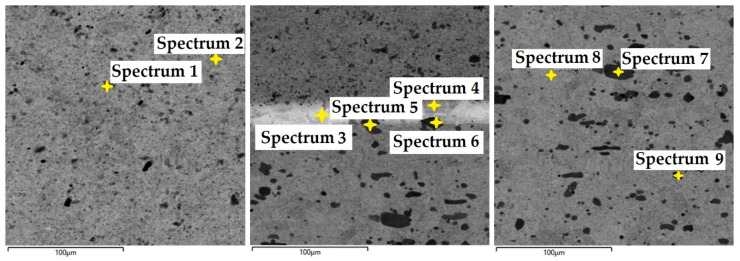
EDS analysis of a laminated steel sample consisting of K110 steel (core steel) and N695 steel with a nickel interlayer.

For evaluating the chemical composition of the individual structural components, point EDS analysis was employed (Figure 29). The results are presented in Table 5, which shows the element content in wt. % and the measurement error of ±3σ. SEM observations revealed the difference in carbide morphology between the edge steel and the core steel. A comparable characterization of laminated tool steels has shown that the nickel interlayer plays a critical role in bonding and diffusion across the interface between dissimilar steels. SEM and EDS techniques are particularly effective in distinguishing carbide morphology and phase boundaries in martensitic matrices with varying alloy content [19]. Similar investigations into multi-material tool steels with nickel-based interlayers have demonstrated that the Ni-rich layer not only ensures metallurgical bonding between dissimilar alloys, but also acts as a diffusion buffer—modulating elemental gradients and phase transformations across the transition zone. SEM and EDS analyses effectively capture the distinct carbide morphologies and compositional shifts in martensitic regions adjacent to the interlayer. Notably, differences in thermal histories and carbon content of the adjoining steels contribute to the formation of heterogeneous carbide populations [19], which in turn influence toughness and hardness gradients in the laminated structure.

**4**. **Forge-Welded Damascus Steel K600 and K720**

**Figure 30:**
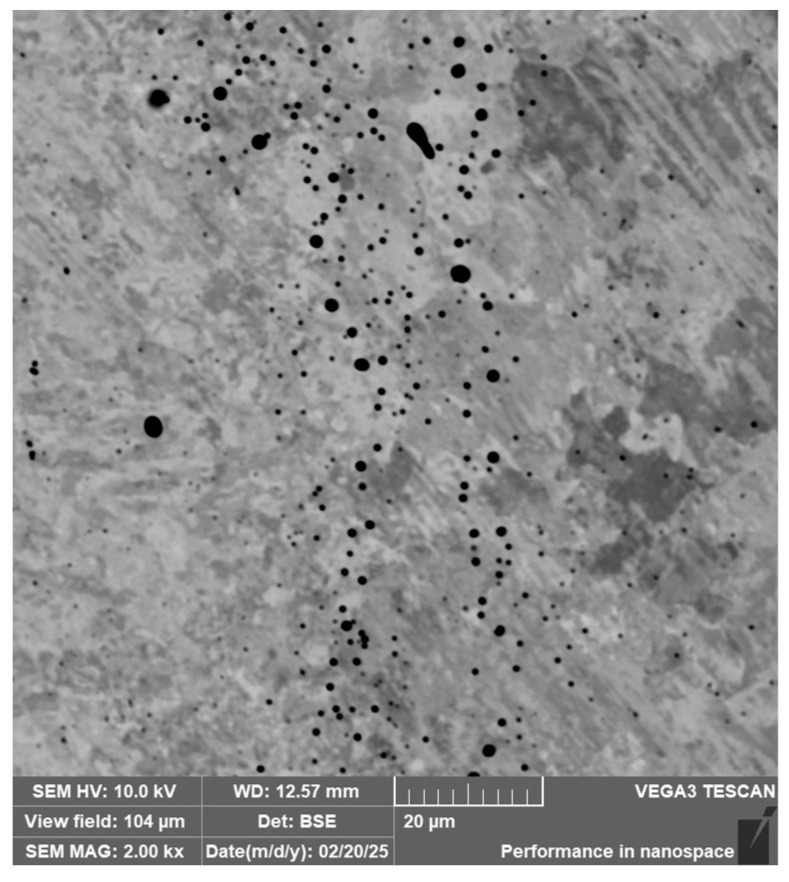
SEM of a sample of forge-welded Damascus steel K600 and K720, area of the forge weld.

**Figure 31:**
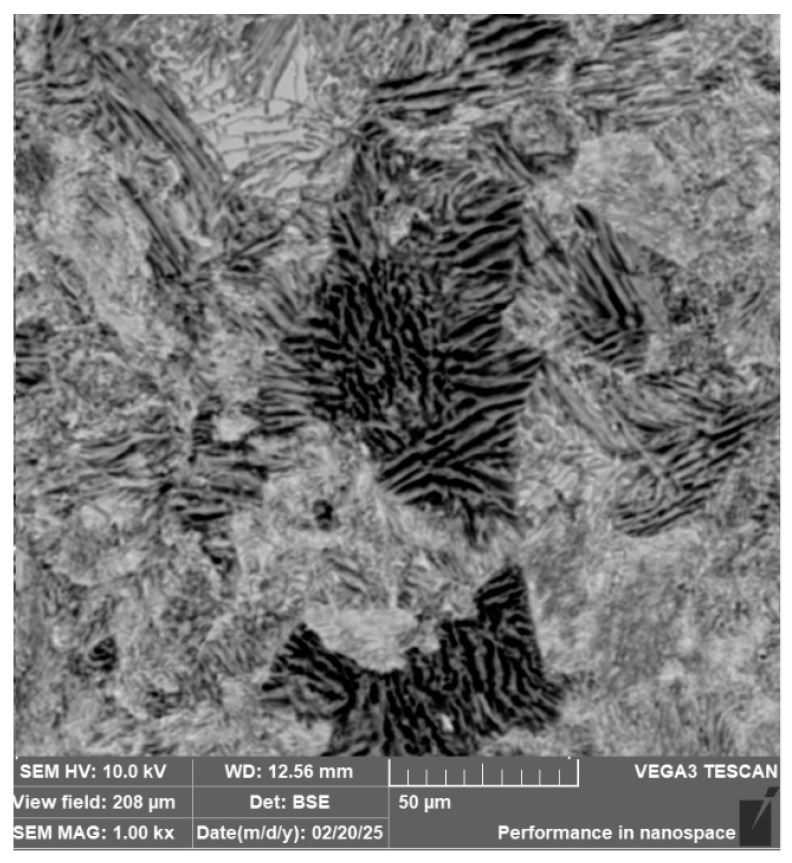
SEM microstructure of K600 steel.

**Figure 32:**
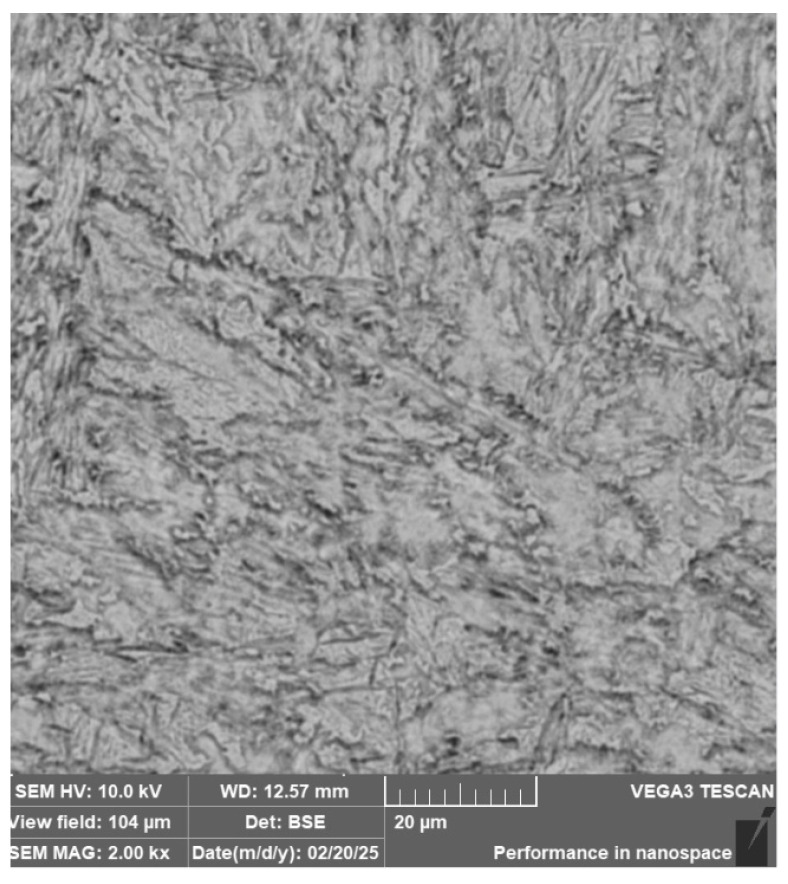
SEM microstructure of K720 steel.

**Figure 33:**
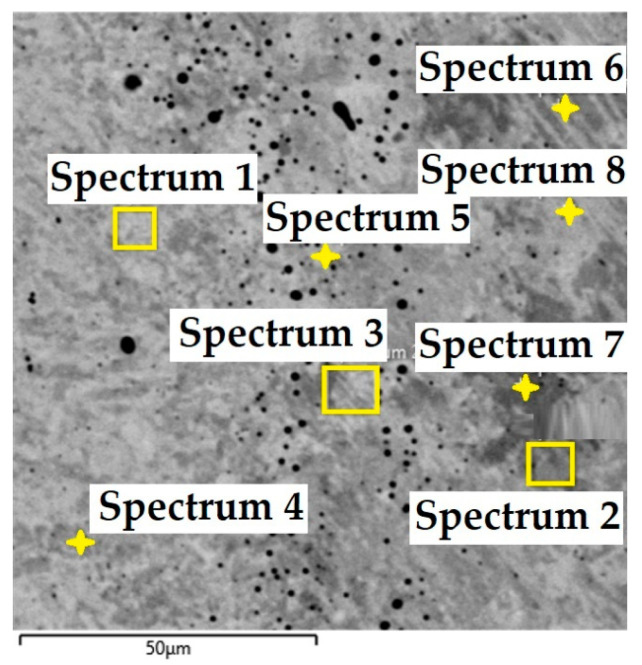
EDS analysis of a sample of forge-welded Damascus steel K600 and K720.

In the SEM and EDS analysis, three areas of the sample with different microstructures were observed. These included the forge weld zone (Figure 30). Figure 31 illustrates the ferritic–pearlitic microstructure of K600 steel, while Figure 32 displays the microstructure of K720 steel. For EDS analysis, both point and area analyses were used (Figure 33). In this sample, it was possible to focus on larger regions of the microstructure, since it was not necessary to measure the chemical composition of individual carbides, as in the previous samples. Gas bubbles were found in the forge weld zone. The results of the EDS analysis are presented in Table 6, which provides the element content in wt. % and the measurement error of ±3σ. The use of SEM in combination with EDS enables a detailed examination of microstructural heterogeneities and localised inclusions or gas voids that may form during forging or welding processes. This methodology is effective in characterising weld zones, identifying inclusion morphology, and analysing elemental segregation in steels with complex thermal histories [20].

In the VG10 steel sample, three areas with different microstructures were identified. One of these areas consisted of 31 layers of stainless steel and nickel, i.e., laminated, folded stainless steel, which morphologically appears under the microscope as “bricks” with an inserted nickel interlayer. In this case, nickel was added not as a carbon diffusion barrier but to enhance the aesthetic appearance of the final blade. In the transition layer between the laminated steel and the core, carbides precipitated along grain boundaries were observed. In the core VG10 steel microstructure, both fine, regularly dispersed carbides and large, irregularly shaped carbides were found, which will affect the mechanical properties of the final knife. The precipitation of both carbide types was caused by an unsuitable heat treatment regime, either due to excessively high temperature or excessively long holding at the hardening temperature. Other research has reached similar conclusions [19]. In this case, the heat treatment regime must be adjusted to obtain a homogeneous microstructure with fine, regular carbides in the VG10 core and to limit carbide precipitation at grain boundaries to ensure the highest possible blade quality. EDS analysis revealed that in the transition zone and the laminated steel region, chromium carbides precipitated with a composition of Fe 81.7 ± 9.9 wt. %, Cr 15.3 ± 1.7 wt. %, C 3.0 ± 1.6 wt. % (Spectrum 1, 2, Figure 23). In the laminated interlayer, Ni was identified at 1.45 ± 1.1 wt. % (Spectrum 8, 9, Figure 23). In the VG10 core, chromium carbides were identified with a composition of Fe 26.2 ± 0.5 wt. %, Cr 55.6 ± 0.5 wt. %, C 12.9 ± 0.2 wt. %, Mo 3.0 ± 0.2 wt. %, and V 2.3 ± 0.2 wt. % (Spectrum 16, Figure 23).

The RWL_34_^TM^ steel sample exhibited a microstructure free of material defects. This steel features a fine martensitic microstructure with uniformly distributed carbides throughout the entire material. This is typical for powder-metallurgical tool steels with optimized hardenability and carbide control [21]. This type of Damascus steel is particularly suitable for knife production. SEM analysis of the microstructure complemented and confirmed the results of optical light microscopy. EDS analysis confirmed the presence of fine (Cr,Mo,V)C carbides with a chemical composition of Fe 37.6 ± 0.5 wt. %, Cr 40.2 ± 0.4 wt. %, C 9.5 ± 0.2 wt. %, Mo 11.9 ± 0.3 wt. %, and V 0.7 ± 0.2 wt. % (Spectrum 1–3, Figure 25).

The laminated steel sample, composed of K110 (core) and N695 with a nickel interlayer, consisted of three distinct regions from a microstructural perspective. The K110 core exhibited a martensitic matrix with primary coarse carbides and fine secondary carbides. Ideally, fine chromium carbides should be present in the martensitic structure of the core steel. However, in this case, large, irregularly distributed carbides were observed in the structure, some of which lacked cohesion with the martensitic matrix [19]. This will affect the mechanical properties of the steel, particularly its strength. The excessive growth of carbides in N695 steel was caused by an improper heat treatment regime, either due to excessively high hardening temperature or prolonged holding time. EDS analysis identified (Cr, V, Mo)C carbides in the K110 core with a composition of Fe 35.7 ± 0.5 wt. %, Cr 44.3 ± 0.45 wt. %, C 13.3 ± 0.2 wt. %, V 4.9 ± 0.2 wt. %, and Mo 1.9 ± 0.2 wt. % (Spectrum 7–9, Figure 29). In the primary carbides of N695 steel, the composition was Fe 28.0 ± 0.5 wt. %, Cr 60.4 ± 0.5 wt. %, and C 11.6 ± 0.2 wt. % (Spectrum 1, Figure 29). These primary carbides contained no V or Mo. In the nickel interlayer, the elements Fe 17.1 ± 0.8 wt. %, Cr 3.45 ± 0.35 wt. %, C 3.6 ± 0.2 wt. %, and Ni 75.9 ± 0.9 wt. % were identified (Spectrum 3, 4, Figure 29).

K720 steel is a type of tool steel, in which a martensitic structure with fine carbides can be expected after heat treatment. However, in this sample, a tempered bainitic microstructure was observed, indicating slow cooling from the hardening temperature. In K600 steel, a ferritic–pearlitic structure was observed, typical of carbon tool steels after insufficient hardening. This means that the cooling rate was not fast enough, leading to a diffusive transformation of austenite, resulting in ferrite+pearlite. In the transition zone, i.e., the forge weld region, microstructural defects (bubbles) were identified. Similar microstructural inhomogeneities have been described in studies of dissimilar tool steel welding and LDED fabrication, where improper cooling leads to non-martensitic structures and porosity at interfaces [19,21]. EDS analysis identified elements defining both steel types. For K720 steel, the composition was Fe 93.8 ± 1.05 wt. %, Cr 1.5 ± 0.2 wt. %, C 3.85 ± 0.2 wt. %, Ni 2.9 ± 1.1 wt. % (Spectrum 1, 4, Figure 33). K600 steel contained Fe 94.8 ± 0.4 wt. %, C 3.05 ± 0.22 wt. %, and Mn 1.65 ± 0.3 wt. % (Spectrum 2, 6, 7, 8, Figure 33). In the forge weld region, the following composition was measured: Fe 94.9 ± 0.4 wt. %, Cr 0.95 ± 0.2 wt. %, C 3.35 ± 0.2 wt. %, Ni 1.6 ± 1.1 wt. % (Spectrum 5, 3, Figure 33).

### 3.3. Microhardness Measurement

Microhardness testing focused on the evaluation of the hardness of individual structural layers of the steels, which were observed and documented in Section 3.2.1 and Section 3.2.2. Each microstructure, as part of a single sample, had a different number of measurements under the same load, depending on the composition in terms of the layers/structures of which the knife steel was made. For each such layer or structure, 10 measurements were performed. The samples were measured in automatic mode, i.e., measurement patterns were set using the 1000× objective. For linear interlayers, a single-line pattern was applied with an indentation spacing of 50 µm. For other structures, a zig-zag pattern was chosen (indentation spacing of 50 µm with an amplitude of 50 µm) to cover a sufficiently large area of the sample and to obtain a realistic representation of the microhardness of the individual microstructures within each tested sample. The intention was not to avoid carbide particles during measurement, as these are an integral part of steel microstructures. This led to higher deviations in some samples, as the indenter occasionally hit carbide particles, which resulted in locally higher hardness values.

**1**. **VG10 Steel with Stainless Steel Edges Laminated with Nickel and a VG10 Steel Core**

**Figure 34:**
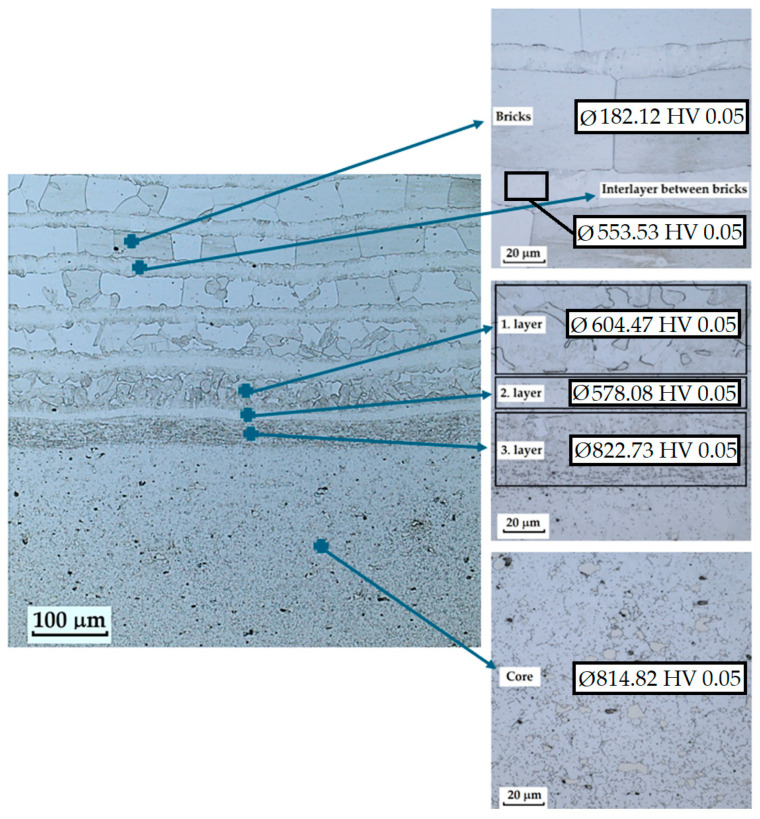
Measured layers of a VG10 steel sample with edges laminated with stainless steel and low alloy steel with a VG10 steel core.

In this sample, six layers or structural regions were identified, as indicated in Figure 34. These images are provided for illustration to clarify how the measurement regions were defined.

Microhardness testing showed no significant differences in hardness among the individual bricks. The hardness in the brick’s region reached an average of 182.12 ± 6.68 HV 0.05. This region also exhibited the greatest indentation depth, approximately. 7.7 µm (compared with approx. 6.3 µm for the others), confirming that the bricks region was the softest in the entire composite. The interlayer between bricks showed higher variability, with an average hardness of 553.53 ± 33.69 HV 0.05. Furthermore, three interlayers were measured: the first interlayer had a hardness of 604.47 ± 57.57 HV 0.05, the second interlayer 578.08 ± 41.56 HV 0.05, and the third interlayer 822.73 ± 19.38 HV 0.05. The VG10 steel core itself exhibited a hardness of 814.82 ± 75.25 HV 0.05.

**2**. **RWL_34_^TM^ Steel (Produced by Powder Metallurgy)**

**Figure 35:**
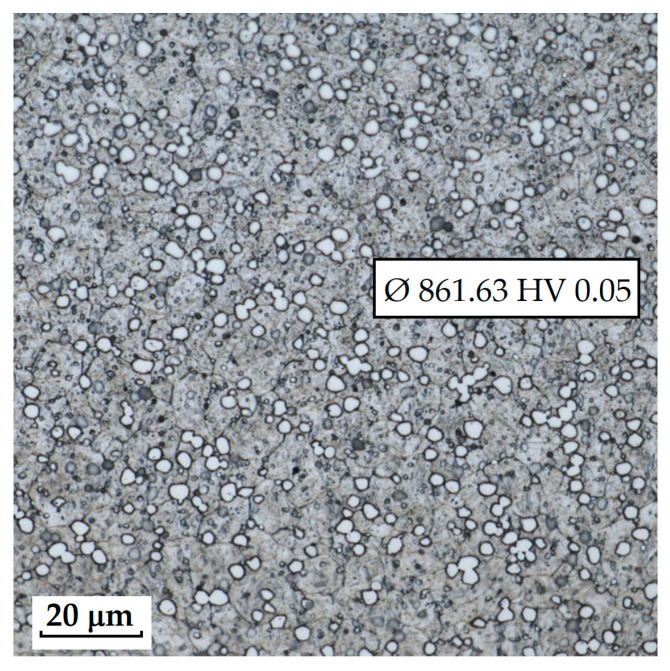
Measured martensitic stainless RWL_34_^TM^ steel with uniformly distributed spherical carbides.

The sample of this steel reached an average microhardness of 861.63 ± 27.16 HV 0.05. In this microstructure (see Figure 35), no layers were identified, only fine, uniformly distributed spherical carbides.

**3**. **Laminated Steel Composed of K110 (Core Steel) and N695 with a Nickel**

**Figure 36:**
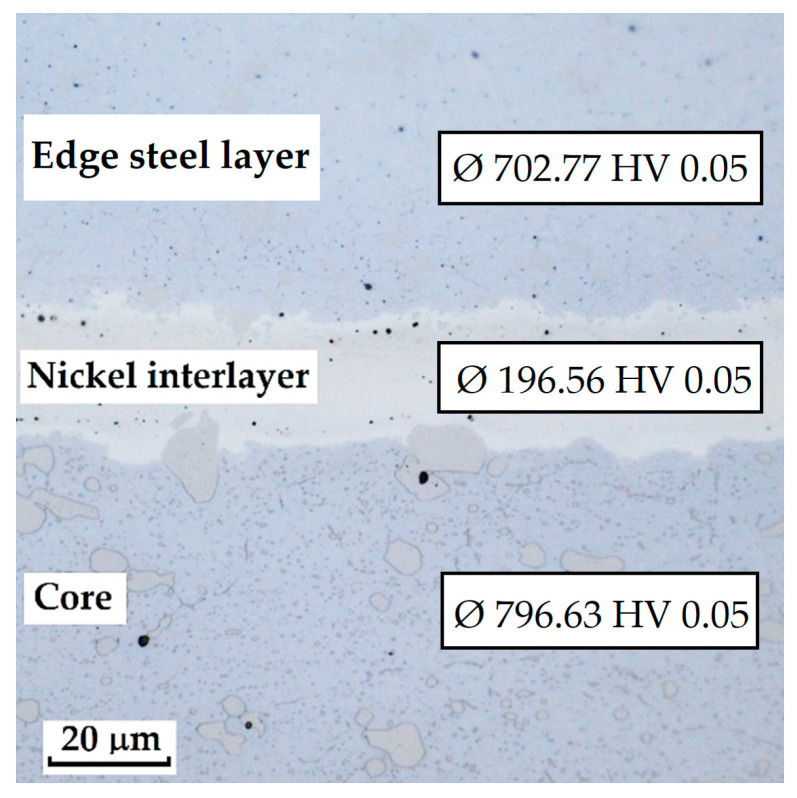
Measured layers of a laminated steel sample consisting of K110 steel (core steel) and N695 steel with a nickel interlayer.

As this is a laminated steel, three regions (layers) were identified and measured in this sample, as shown in Figure 36.

Each layer exhibited a different microhardness value. The nickel interlayer reached an average hardness of 196.56 ± 8.74 HV 0.05. The N695 layer showed 702.77 ± 32.33 HV 0.05, and the core achieved 796.63 ± 22.40 HV 0.05. In both the core and the outer steel layer, higher deviations were measured due to the presence of large carbides in the steel microstructure.

**4**. **Forge-Welded Damascus Steel K600 and K720**

**Figure 37:**
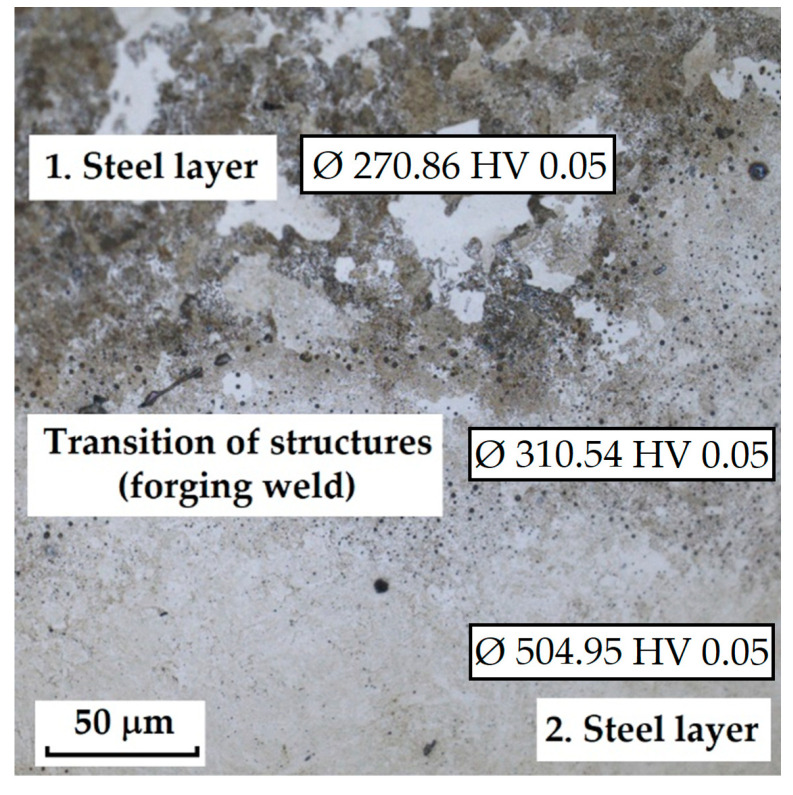
The measured layers of the sample were forge-welded Damascus steel K600 and K720.

In this knife steel sample, three structures were identified (Figure 37). The first steel layer (ferritic–pearlitic structure) reached an average microhardness of 270.86 ± 10.68 HV 0.05. The transition zone between the steel structures reached 310.54 ± 38.25 HV 0.05, and the second steel layer (tempered bainite) achieved 504.95 ± 29.71 HV 0.05.

The results of microhardness measurements (Table 7) of the individual layers and structures confirmed the significant influence of material heterogeneity on the hardness profile of the studied steels. In the VG10 sample, pronounced differences were measured between the regions, where the laminated layers (Bricks) exhibited the lowest values (~182 HV 0.05), while the VG10 core steel reached hardness above 800 HV 0.05. Deviations within the layers were partly caused by the presence of large carbides, which locally increased the indentation values. RWL_34_^TM^ steel, produced by powder metallurgy, showed a homogeneous microstructure without significant deviations and high hardness (~862 HV 0.05), corresponding to its fine martensitic state. The laminated steel K110+N695 achieved higher hardness in the core layer (K110) compared to the edge layer (N695), while the nickel interlayer had significantly lower hardness (~197 HV 0.05), consistent with its composition and function. In the forge-welded Damascus steel K600+K720, the difference between the ferritic–pearlitic region (~271 HV 0.05) and the tempered bainitic region (~505 HV 0.05) was confirmed, with the transition zone showing intermediate values. These results demonstrate that the hardness of individual layers is closely related to their phase composition, production technology, and heat treatment parameters. In contrast, the presence of carbides and layer interfaces may cause local deviations.

### 3.4. Material Defects and Structural Inhomogeneities

In the evaluation of the microstructure of individual samples, material defects and structural inhomogeneities were identified, which did not affect the final appearance of the manufactured knives but may influence their mechanical properties (particularly strength) during use. In the structures of the steels we examined, two main types of material defects and structural inhomogeneities were observed: excessively large carbides, carbides at grain boundaries and carbide banding, and gas bubbles and lack of bonding in the transition zones of the steels (welds between structures), which are documented in the following figures.

**1**. **Large Carbides, Carbides at Grain Boundaries, and Carbide Banding**

**Figure 38:**
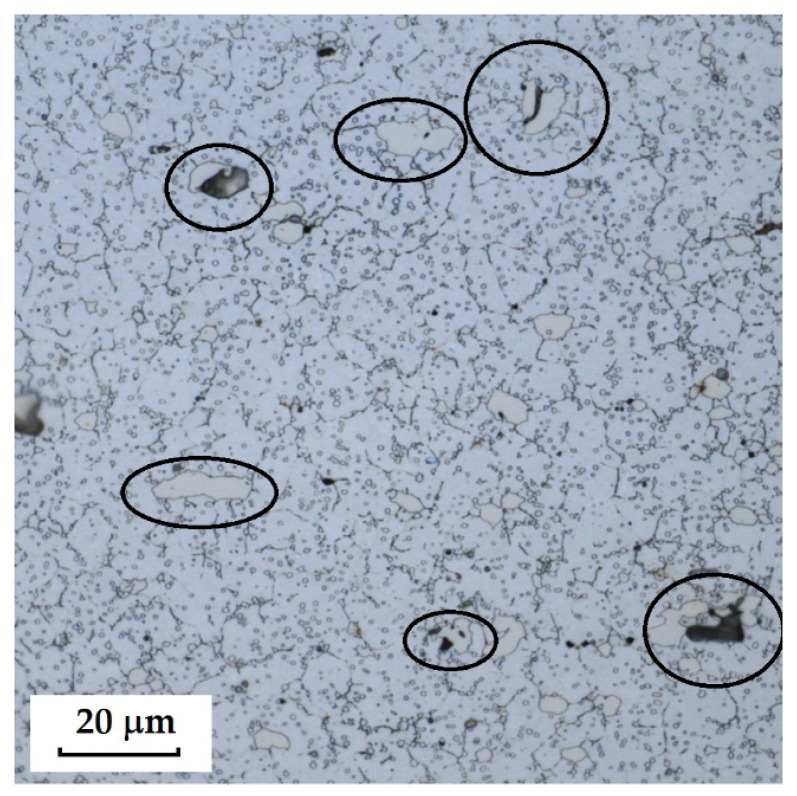
Marked large carbides in VG10 core steel.

**Figure 39:**
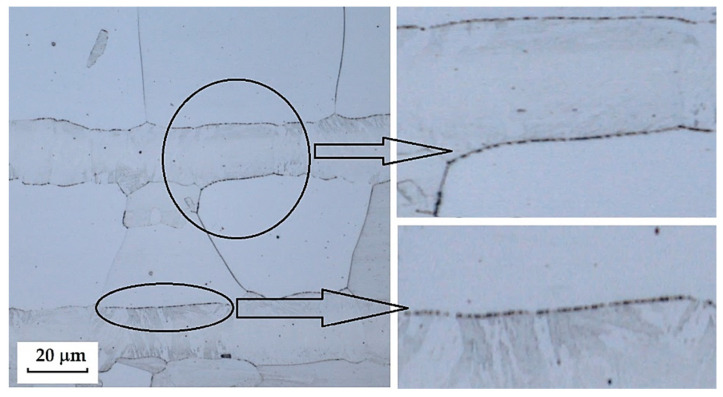
Carbide exclusion at grain boundaries/Bricks and interlayer boundary.

**Figure 40:**
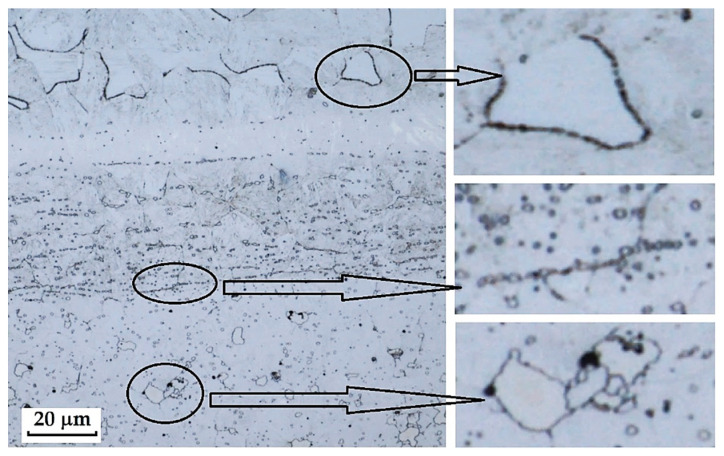
Exclusion of carbides in the transition layer (carbides at the grain boundary, carbide streaking, large carbides in the core steel).

The VG10 steel sample contained carbides that precipitated both at grain boundaries—predominantly in the transition zone between the laminated steel structure and the core—as well as within the core microstructure itself. In the core steel, large carbides were found, and in some cases, fracture of these carbides was observed. Around these large carbides, cracks and voids form, which under mechanical loading may act as crack initiators and ultimately lead to steel fracture. Figure 38 documents and marks the large carbides, where carbide fracture and adjacent cracks/voids can be observed. Figure 39 and Figure 40 show carbide precipitation at grain boundaries. Carbides at grain boundaries precipitated not only in the transition zone between the laminated part of the sample and the core, but also at the boundaries of the individual Bricks and the interlayer. In the microstructure of the transition zone, carbide banding formed by fine carbides can also be observed. That these features were indeed carbides was confirmed by EDS analysis.

**Figure 41:**
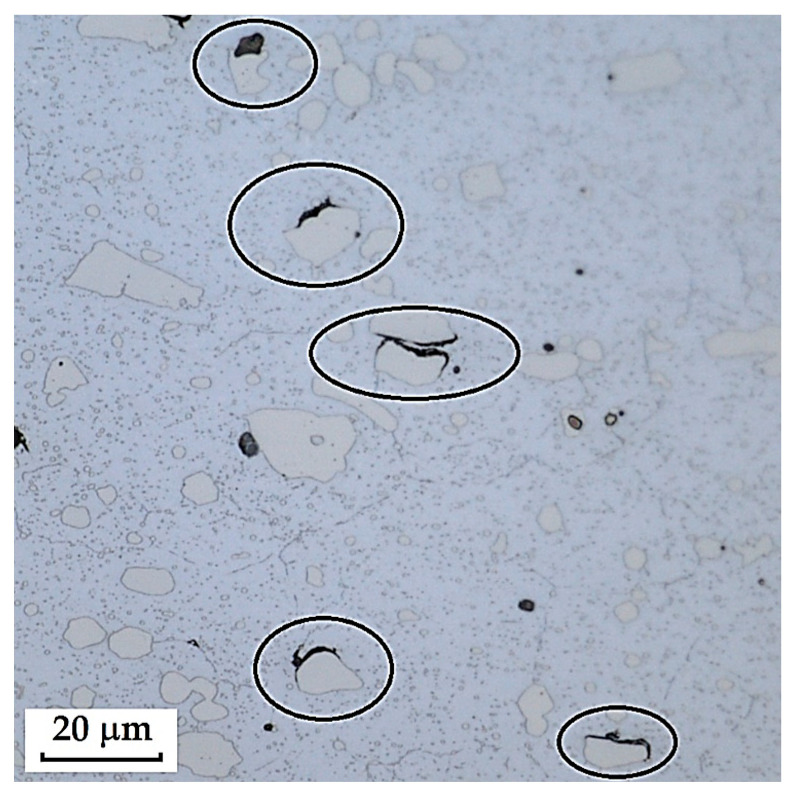
Exclusion of large carbides in the K110 core steel, violation of cohesion between the steel and carbides.

The laminated steel, composed of K110 (core steel) and N695 with a nickel interlayer, exhibited defects in its core in the form of large, irregularly distributed carbides in the martensitic matrix. Figure 41 shows the separation of carbides from the matrix and the formation of voids, which could act as initiators of crack formation.

**2**. **Porous Cavities and Lack of Bonding in the Transition Zones of Steel (Structural Welds)**

**Figure 42:**
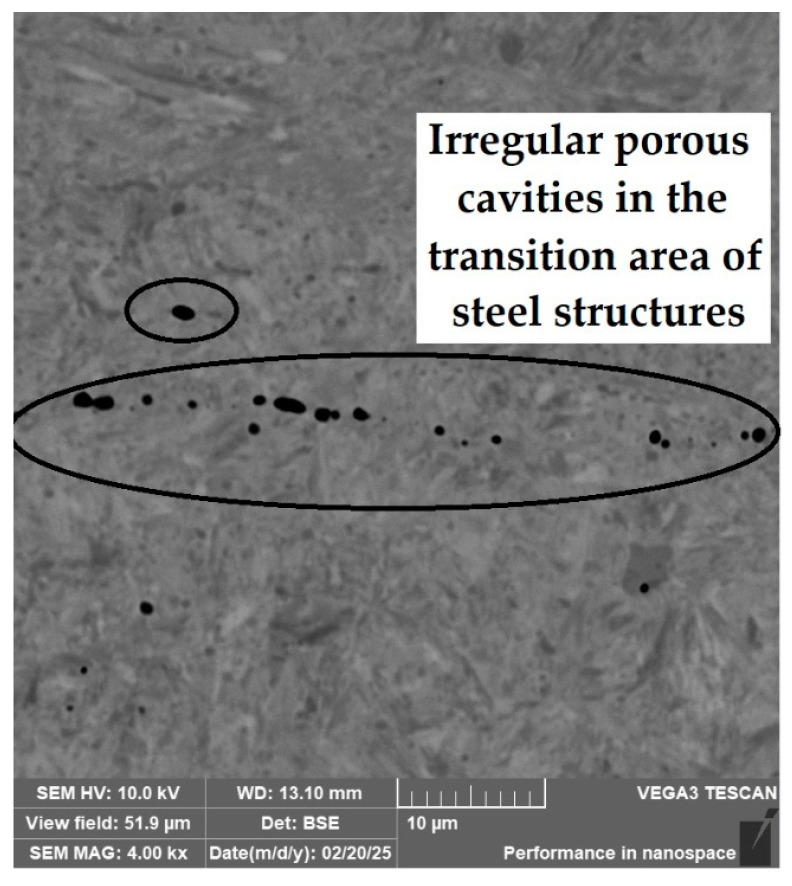
Irregular porous cavities in the transition region of a VG10 steel sample.

**Figure 43:**
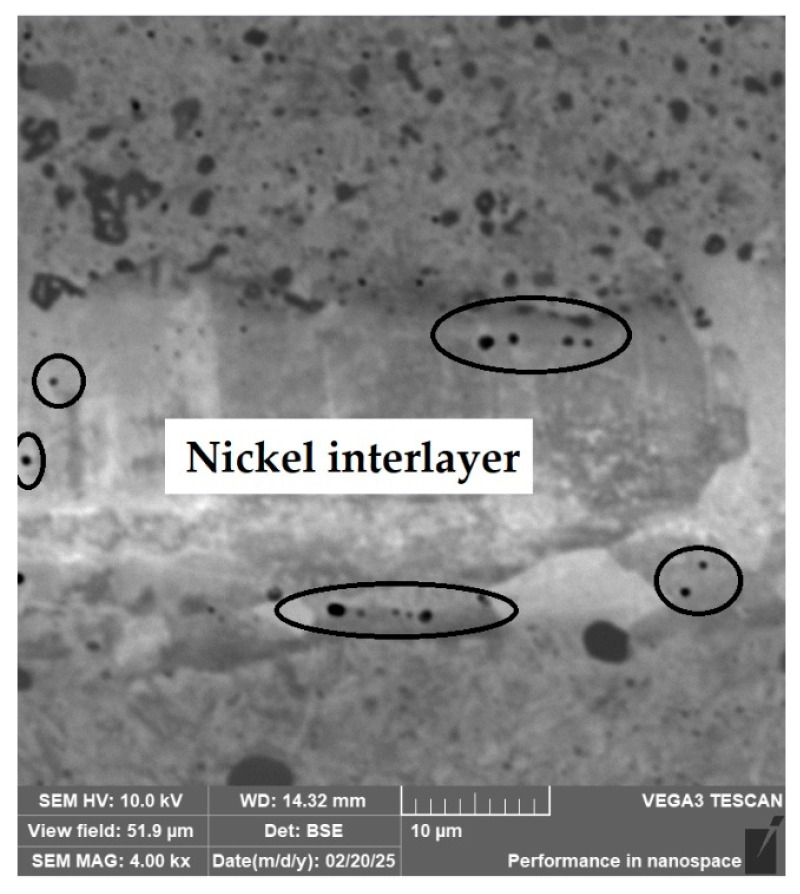
Irregular porous cavities in the transition region of the nickel interlayer of the K110 and N695 steel samples.

In three samples, microscopic analysis revealed irregular porous cavities in the weld zone and transition structures. This structural defect was most evident in the sample of forge-welded Damascus steel made from K600 and K720 steels (Figure 30, Section 3.2.2). A very similar defect was also observed in the VG10 sample between the core and the folded edge steel (Figure 42). The laminated steel samples K110 and N695 exhibited a comparable defect at the edges of the nickel interlayer (Figure 43).

**3**. **Growth of Austenitic Grains After Heat Treatment**

**Figure 44:**
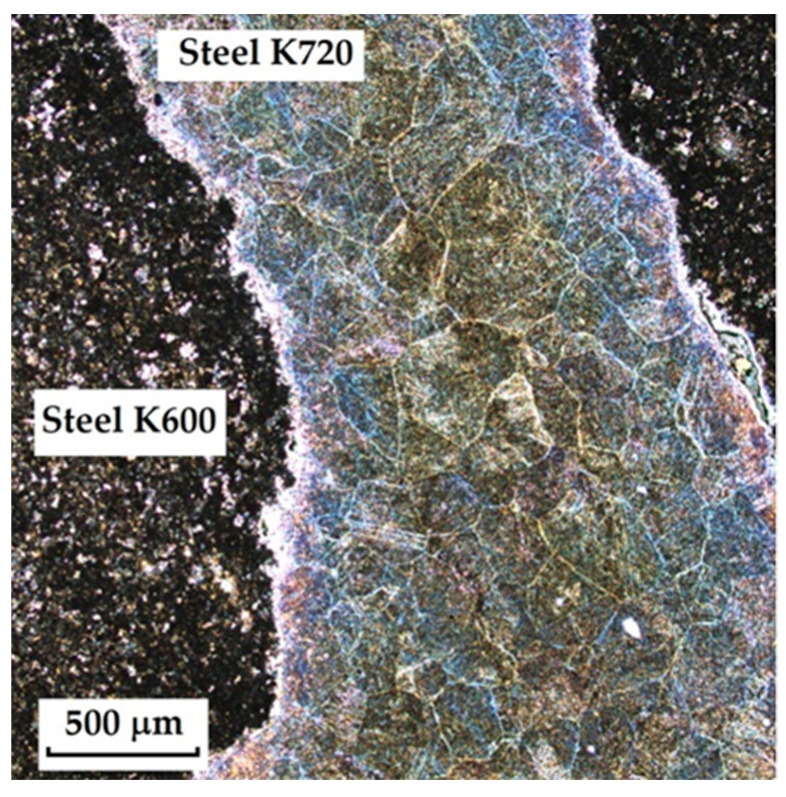
Large original austenitic grains in the structure of K720 steel.

Although the microstructure in Figure 7 (Section 3.2) was evaluated as heavily overetched, particularly in the K720 steel region, one important finding was obtained. In the K720 steel, the boundaries of the original austenitic grains were revealed by etching. The image shows that grain growth had occurred. Figure 44 clearly displays the boundaries of these grains, indicating incorrectly chosen heat treatment conditions (excessively long holding time at the hardening temperature).

Microstructural analysis revealed several types of defects and inhomogeneities that may negatively affect the mechanical properties and service life of the final blades. The most frequently observed defects were excessively large carbides, particularly typical for the core layers of VG10 and K110 steels. These carbides exhibited loss of cohesion with the matrix and the presence of voids in their vicinity, which may lead to crack initiation under mechanical loading. Another defect identified was the presence of porous cavities and a lack of bonding in the transition zones of layers, most pronounced in the forge-welded Damascus steel K600+K720, as well as in the laminated steels VG10 and K110+N695. These defects most likely originated from insufficient fusion of the layers during welding or from inappropriate forming regimes. In K720 steel, excessive growth of the original austenitic grain size was also recorded, indicating an excessively long holding time at the hardening temperature. The presence of these defects confirms that, to achieve optimal mechanical properties, it is essential not only to select the appropriate chemical composition and production technology, but also to optimise the parameters of heat treatment and joining operations.

The research presented provides new insights into the microstructural characterisation and mechanical evaluation of modern multilayer knife steels.

By combining optical and electron microscopy (SEM), EDS analysis, and microhardness measurements, the behaviour of four types of composite steels—VG10, RWL_34_^TM^, K110+N695, and K600+K720—was thoroughly analysed. The results showed that standard chemical-composition analysis methods (OES) are insufficient for multilayer materials and must be complemented by EDS, which enables the separate measurement of individual layers, including transition zones.

A key finding is the effect of improperly selected heat-treatment parameters on the formation of large carbides in the core steels VG10 and K110, which were observed both within the matrix and along grain boundaries. This condition weakens the structure and represents potential sites for crack initiation, as also reported by Moheimani et al. [22] who described the detrimental influence of carbides on toughness in the tested steels. In contrast, the powder-metallurgical steel RWL_34_^TM^ exhibits excellent microstructural homogeneity and high hardness, making it suitable for applications requiring superior edge retention and cutting performance. Similarly, Huang et al. [23] confirmed the importance of microstructural control during layer bonding in multiphase materials.

A novel contribution of this study is the identification of the significant role of the nickel interlayer between individual steels—not only for aesthetics, but primarily as a diffusion barrier and a stress-relieving element between layers. In practice, this means that the composition and thickness of the Ni interlayer can either improve or impair the blade’s resistance to delamination.

The transition regions between materials are also critical sites for defect formation (porosity, discontinuities), which aligns with the findings of Sun et al. [24] for multiphase stainless steels with bainitic structures. Microhardness results further confirm the relationship between phase composition and the local mechanical resistance of individual layers.

This work provides a comprehensive comparison of structurally and technologically distinct types of knife steels. It offers practical recommendations for selecting appropriate heat-treatment regimes and diagnosing hidden defects in real blade production. A unique contribution of the study is the systematic comparison of four types of knife steels: powder metallurgical (RWL_34_^TM^), forged (K600+K720), laminated (K110+N695), and pre-laminated (VG10).

By combining optical microscopy, SEM/EDS analysis, and microhardness testing, the study offers a comprehensive perspective on the behaviour of multilayer composites, which remain underexplored in the current literature. The obtained results have direct practical relevance not only in the field of cutlery. They highlight the need to reassess heat-treatment regimes, note the unsuitability of universal etching agents, provide guidelines for detecting defects (e.g., pores or coarse carbides), and present hardness data for individual layers—key for grinding processes and achieving optimal blade sharpness.

## 4. Conclusions

The combination of advanced analytical methods, including optical and electron microscopy, EDS, and microhardness testing, enabled a detailed examination of the interactions between microstructure, heat treatment processes, and the quality of the final blades, thereby contributing to the optimisation of manufacturing procedures. The study demonstrated that the quality and functionality of special knife steels are closely linked to the choice of base material and to the heat treatment regime. Microscopic, and especially SEM/EDS analysis, confirmed the decisive role of carbide morphology and distribution, interlayer cohesion, and the homogeneity of phase boundaries.

Powder steel RWL_34_^TM^ proved to be a stable system with a fine-grained martensitic matrix and uniformly dispersed (Cr,Mo,V)C carbides, ensuring high hardness (~862 HV 0.05) and the absence of structural defects. This result confirms the superiority of powder metallurgy and establishes it as a reference standard to produce high-performance blades.

In contrast, laminated steels (VG10, K110+N695) revealed the presence of large primary carbides, segregations, and porous cavities, particularly in the nickel interlayer regions. These defects represent potential sites for crack initiation and reduce toughness. Nevertheless, laminated systems offer an attractive compromise between functional properties and aesthetic value of the blade, making them suitable for applications where design is equally important as performance.

Forge-welded Damascus steel K600+K720 exhibited a heterogeneous structure combining ferritic–pearlitic and bainitic matrices, along with weld defects and gas bubbles. These shortcomings indicate suboptimal welding and quenching conditions. However, the results also suggest that traditional forging techniques can be promising when optimised and carefully controlled.

The research confirmed the dominant influence of heat treatment parameters—austenitizing temperature, holding time, and cooling rate—on the resulting microstructure and mechanical properties of the blades. Optimal regimes lead to homogeneous martensitic matrices with fine carbides, while inappropriate conditions promote coarse carbides, segregation, and defects.

The study demonstrates that the synergy of traditional technologies (lamination, forge welding) with modern approaches (powder metallurgy, controlled heat treatment) paves the way to produce knives that surpass traditional limits in both functionality and aesthetics. These findings expand current scientific literature and provide practical guidance for the design and application of high-performance knife steels in materials engineering.

Key findings:

- **RWL_34_^TM^:** Homogeneous martensitic microstructure with finely dispersed (Cr,Mo,V)C carbides; high hardness (~862 HV 0.05) and absence of structural defects.
- **VG10:** Large carbides at grain boundaries and in the core, matrix caused by an unsuitable heat treatment regime; presence of Ni in interlayers.
- **K110+N695:** Composite with a nickel interlayer; large primary carbides are present in the core, and fine secondary carbides are present in the matrix. The interlayer (~75 wt. % Ni) contributes to aesthetics but represents a defect-sensitive zone.
- **K600+K720:** Heterogeneous microstructure (ferritic–pearlitic and bainitic matrices), weld defects and bubbles; SEM revealed growth of the original austenitic grains.
- **SEM/EDS:** An expensive but essential tool for evaluating the quality of knife steels, enabling direct observation of morphology, chemical composition, and defective regions.

## Figures and Tables

**Table 1:** Chemical composition of steels—manufacturer vs. measured [wt. %].

Steel/Element	C	Mn	Si	Cr	Mo	Co	Ni	V	P	S
Chemical composition declared by the steel manufacturer										
VG10	1.00	0.50	0.60	15.00	1.05	1.40	-	0.20	0.30	-
RWL_34_^TM^	1.05	0.50	0.50	14.00	4.00	-	-	0.20	-	-
K110	1.55	0.35	0.25	11.80	0.80	-	-	0.95	-	-
N695	1.08	max. 1.00	max. 1.00	17.00	0.60	-	-	-	max. 0.04	max. 0.02
K600	0.45	0.40	0.25	1.30	0.25	-	4.00	-	-	-
K720	0.90	2.00	0.25	0.35	-	-	-	0.35	-	-
Chemical composition—measured (OES TASMANN Q4, average from 6 measurements each sample)										
VG10	0.975	0.278	0.441	17.15	<0.0100	0.011	0.154	0.028	<0.0050	<0.150
RWL_34_^TM^	0.053	0.507	0.802	15.50	0.085	0.023	0.150	0.123	<0.0050	<0.150
Laminate K110+N695	0.967	0.303	0.375	13.36	3.788	0.014	0.089	0.199	<0.0050	<0.150
Forge welded K600+K720	0.534	0.336	0.367	1.543	0.298	<0.0050	4.005	0.017	0.334	<0.150

**Table 2:** Etching of metallographic samples of steels.

Etchant/Sample	Steel VG10	Steel RWL_34_^TM^	Laminate K110+N695	Forge Welded K600+K720
Kroll/time	1 min 15 s	10 s	1 min	3 s
Result Kroll	NOK	OK	NOK	NOK
Nital 3%/time	2 min	2 min	2 min	20 s
Result Nital 3%	NOK	NOK	NOK	NOK
Nital 3%/time	4 min	4 min	4 min	50 s
Result Nital 3%	NOK	NOK	OK	OK
V2A (20 °C)/time	1 min 30 s	1 min 30 s	45 s	10 s
Result V2A	OK	OK	NOK	NOK

**Table 3:** EDS sample VG10 analysis [wt. %].

Spectrum	Description of Structure Components	Element					
		Fe	Cr	C	Ni	Mo	V
1	Grain boundary carbide	82.09 ± 10.34	14.75 ± 1.81	3.17 ± 1.93	-	-	-
2	Grain boundary carbide	81.32 ± 9.59	15.91 ± 1.73	2.87 ± 1.36	-	-	-
3	Laminated stainless steel	79.4 ± 0.4	17.7 ± 0.4	2.9 ± 0.2	-	-	-
4	Laminated stainless steel	79.5 ± 0.4	17.8 ± 0.4	2.8 ± 0.2	-	-	-
5	Laminated stainless steel	79.1 ± 0.4	18.1 ± 0.4	2.8 ± 0.1	-	-	-
6	Laminated stainless steel	79.2 ± 0.4	17.9 ± 0.4	2.9 ± 0.2	-	-	-
7	Laminated stainless steel	79.0 ± 0.4	18.3 ± 0.4	2.8 ± 0.2	-	-	-
8	Steel interlayer with nickel content	77.4 ± 0.1	17.8 ± 0.4	3.4 ± 0.2	**1.4 ± 1.1**	-	-
9	Steel interlayer with nickel content	78.2 ± 1.0	17.3 ± 0.4	3.1 ± 0.2	**1.5 ± 1.1**	-	-
10	Laminated stainless steel	79.8 ± 0.4	17.6 ± 0.4	2.6 ± 0.1	-	-	-
11	Laminated stainless steel	79.6 ± 0.4	17.7 ± 0.4	2.7 ± 0.1	-	-	-
12	Laminated stainless steel	79.7 ± 0.4	17.8 ± 0.4	2.5 ± 0.1	-	-	-
13	Transition zone	77.8 ± 0.4	**19.1 ± 0.4**	3.1 ± 0.1	-	-	-
14	Transition zone	77.1 ± 0.4	**20.0 ± 0.4**	2.9 ± 0.1	-	-	-
15	Core steel	60.7 ± 0.5	33.3 ± 0.4	6.0 ± 0.2	-	-	-
16	Core steel (carbide)	26.2 ± 0.5	**55.6 ± 0.5**	**12.9 ± 0.2**	-	**3.0 ± 0.2**	**2.3 ± 0.2**
17	Core steel	82.8 ± 0.4	13.9 ± 0.3	3.3 ± 0.2	-	-	-

**Table 4:** EDS sample RWL_34_^TM^ analysis [wt. %].

Spectrum	Description of Structure Components	Element				
		Fe	Cr	C	Mo	V
1	Carbides in martensitic steel matrix	37.4 ± 0.5	40.0 ± 0.4	9.9 ± 0.2	11.7 ± 0.3	0.9 ± 0.2
2	Carbides in martensitic steel matrix	37.5 ± 0.5	40.2 ± 0.4	9.3 ± 0.2	12.3 ± 0.3	0.7 ± 0.2
3	Carbides in martensitic steel matrix	37.8 ± 0.5	40.6 ± 0.4	9.3 ± 0.2	11.7 ± 0.3	0.7 ± 0.2

**Table 5:** EDS analysis of a laminated steel sample consisting of K110 steel (core steel) and N695 steel with a nickel interlayer [wt. %].

Spectrum	Description of Structure Components	Element					
		Fe	Cr	C	Ni	V	Mo
1	Edge layer steel	28.0 ± 0.5	60.4 ± 0.5	11.6 ± 0.2	0.00	0.00	0.00
2	Edge layer steel	81.9 ± 0.4	14.7 ± 0.3	3.4 ± 0.1	0.00	0.00	0.00
3	Nickel interlayer	18.9 ± 0.8	2.9 ± 0.3	3.6 ± 0.2	**74.6 ± 0.9**	0.00	0.00
4	Nickel interlayer	15.3 ± 0.8	4.0 ± 0.4	3.6 ± 0.2	**77.1 ± 0.9**	0.00	0.00
5	Core steel carbide	35.9 ± 0.5	**43.9 ± 0.4**	13.1 ± 0.2	0.00	**4.9 ± 0.2**	**2.2 ± 0.2**
6	Core steel carbide	35.8 ± 0.5	**44.2 ± 0.4**	13.1 ± 0.2	0.00	**5.0 ± 0.2**	**2.0 ± 0.2**
7	Core steel carbide	35.2 ± 0.5	**44.4 ± 0.4**	13.4 ± 0.2	0.00	**4.9 ± 0.2**	**2.0 ± 0.2**
8	Core steel	88.8 ± 0.3	7.8 ± 0.3	3.5 ± 0.2	0.00	0.00	0.00
9	Core steel carbide	36.1 ± 0.5	**44.1 ± 0.5**	13.1 ± 0.2	0.00	**4.9 ± 0.2**	**1.8 ± 0.2**

**Table 6:** EDS analysis of a sample of forge-welded Damascus steel K600 and K720 [wt. %].

Spectrum	Description of Structure Components	Element				
		Fe	Cr	C	Ni	Mn
1	K720 steel	91.0 ± 1.0	1.5 ± 0.2	4.0 ± 0.2	**3.6 ± 1.1**	0.00
2	K600 steel	94.5 ± 0.4	0.00	3.6 ± 0.2	0.00	**1.9 ± 0.3**
3	Forge weld transition area	95.3 ± 0.3	1.1 ± 0.2	3.6 ± 0.2	0.00	0.00
4	K720 steel	92.5 ± 1.1	1.5 ± 0.2	3.7 ± 0.2	**2.2 ± 1.1**	0.00
5	Forge weld transition area	94.4 ± 1.1	0.8 ± 0.2	3.1 ± 0.2	1.6 ± 1.1	0.00
6	K600 steel	94.9 ± 0.4	0.00	3.5 ± 0.2	0.00	**1.5 ± 0.3**
7	K600 steel	94.9 ± 0.4	0.00	1.5 ± 0.3	0.00	**1.5 ± 0.3**
8	K600 steel	94.7 ± 0.4	0.00	3.6 ± 0.2	0.00	**1.7 ± 0.3**

**Table 7:** Results of microhardness measurement.

Measured Sample	Sample Measured Layer	Vickers Microhardness Value HV 0.05	Standard Deviation
VG10 steel with stainless steel edges laminated with nickel and a VG10 steel core	brick’s region	182.12	6.68
	interlayer between bricks	553.53	33.69
	first interlayer	604.47	57.57
	second interlayer	578.08	41.56
	third interlayer	822.73	19.38
	core steel	814.82	75.25
RWL_34_^TM^ steel	-	861.63	27.16
Laminated steel composed of K110 (core steel) and N695 with a nickel	nickel interlayer	196.56	8.74
	N695 layer	702.77	32.33
	core steel	796.63	22.40
Forge-welded Damascus steel K600 and K720	first steel layer	270.86	10.68
	transition zone	310.54	38.25
	second steel layer	504.95	29.71

## Data Availability

The original contributions presented in this study are included in the article. Further inquiries can be directed to the corresponding author.
